# Metallacyclosilanes of Calcium, Yttrium, and Iron

**DOI:** 10.1021/acs.inorgchem.2c02508

**Published:** 2022-10-25

**Authors:** Alexander Pöcheim, Rainer Zitz, Julia Hönigsberger, Christoph Marschner, Judith Baumgartner

**Affiliations:** Institut für Anorganische Chemie, Technische Universität Graz, Stremayrgasse 9, 8010 Graz, Austria

## Abstract

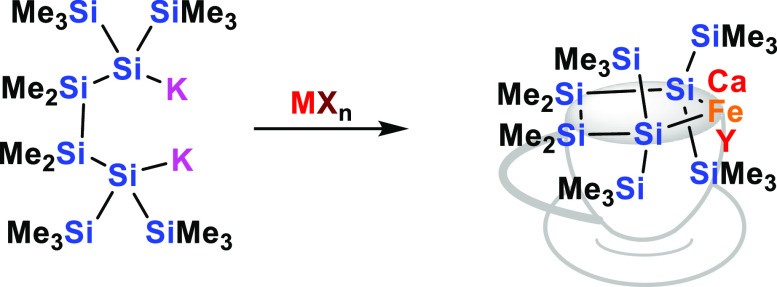

Utilizing a choice of α,ω-oligosilanylene diides, it is possible to synthesize a number of heterocyclosilanes with heteroelements of calcium, yttrium, and iron by metathesis reactions with respective metal halides CaI_2_, YCl_3_, and FeBr_2_. ^29^Si NMR spectroscopic analysis of the calcacyclosilanes suggests that these compounds retain a strong oligosilanylene dianion character, which is more pronounced than in the analogous magnesacyclosilanes. As the electronegativity of calcium lies between potassium and magnesium, silyl calcium reagents should be considered as building blocks with an attractive reactivity profile. Reaction of a 1,4-oligosilanylene diide with YCl_3_ gave the five-membered yttracyclosilane as an ate-complex with two chlorides still attached to the yttrium atom. Reaction of the obtained compound with two equivalents of NaCp led to another five-membered yttracyclosilane ate-complex with an yttracene fragment. When using a dianionic oligosilanylene ligand containing a siloxane unit, the siloxane oxygen acted as an additional coordination site for Ca and Y. When the same ligand was used to prepare a cyclic 1-ferra-4-oxatetrasilacyclohexane, an analogous transannular interaction between the iron and oxygen atoms is missing.

## Abstract 2

Reactions of some α,ω-oligosilanylene diides with CaI_2_, YCl_3_, and FeBr_2_ allow convenient access to metallocyclosilanes with Ca, Y, and Fe as heteroatoms. The calcium and yttrium compounds resemble previously prepared magnesacyclosilanes, retaining a strong silanide character. The only related compounds to the described ferracyclosilanes are acyclic examples.

## Introduction

For a long time, the synthesis of heterocyclosilanes was either restricted to insertion reactions into strained cyclosilanes or to reactions of α,ω-oligosilanylene dihalides with nucleophilic heteroatoms. Preparation of metallacyclosilanes with non-nucleophilic metals, however, was more challenging. The development of a convenient synthetic protocol for α,ω-oligosilanylene dianions^1^ eventually permitted simple access to this class of compounds, where frequently the interaction between the metal and the neighboring silicon atoms is rather polar or at least weaker than typical silicon main-group bonds.

### Silyl Calcium Compounds

One type of compounds with a weak silicon metal interaction are magnesacyclosilanes, which are easily prepared from dipotassium α,ω-oligosilanylene diide compounds by reaction with an equimolar amount of MgBr_2_·Et_2_O.^2−6^ A major reason for our interest in these compounds was to moderate the reactivity of the associated silanides compared to that of dipotassium compounds. Magnesium silanides can be regarded as sila-Grignard-type reagents. Indeed, we could utilize these compounds in a number of reactions where potassium silanides were either too reducing, too basic, or too reactive in general.^7^

Considering organomagnesium (Grignard) compounds, an obvious question is whether instead of magnesium calcium might be used as an alternative metal. A short answer to this question is, “In principle, yes, but these compounds are neither easily prepared as nor can be handled with the same ease as organomagnesium compounds.” Nevertheless, in recent years, the chemistry of organocalcium compounds has been intensively investigated and several synthetically useful methods for their preparation have been developed. The direct (Grignard analogous) synthesis requires activated calcium and preferably organoiodides as starting materials. Other methods include the deprotonation of C–H acidic compounds with calcium amides and transmetallation/metal-exchange reactions.^8−11^

**Chart 1:**
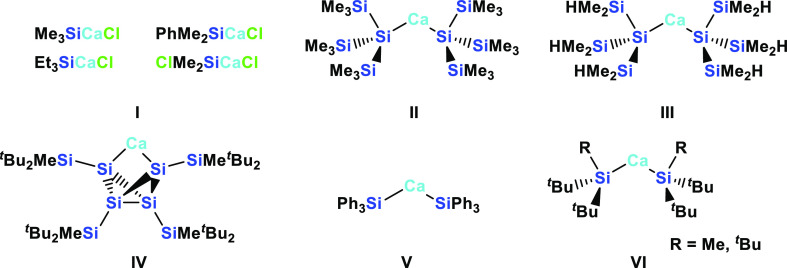
Known Examples of Calcium Silanides **I**,^19^**II**,^20^**III**,^21^**IV**,^22^**V**,^23^ and **VI**(24)

The synthesis of alkaline-earth derivatives of heavier congeners of carbanions, namely, silanides, is associated with similar problems. Although silyl magnesium compounds have gained wide acceptance over the last few years, they are typically not obtained from direct reactions of silyl halides with elemental magnesium^12^ but rather by metathesis of lithium or potassium silanides with magnesium bromide.^13−18^ The same is essentially true for silyl calcium compounds, the number of reported examples still being limited to very few (Chart 1). Remarkably enough, the very first report of calcium silanides by Mochida and Manishi is the only approach that utilizes the direct Grignard-type reaction of insertion of elemental calcium into a Si–Cl bond.^19^ For this purpose, calcium metal vapor was used. Four different silyl calcium compounds (**I**) (Chart 1) were prepared by this method, and based on derivatization reactions with a number of different electrophiles the yields of formation were rather moderate.^19^ All other approaches to calcium silanides reported so far have employed metathesis reactions of lithium or potassium silanides with CaI_2_. Teng and Ruhlandt-Senge demonstrated the principal feasibility of this by reaction of (Me_3_Si)_3_SiK with CaI_2_ to [(Me_3_Si)_3_Si]_2_Ca (**II**) (Chart 1).^20^ A report by the Sadow group on the formation of [(Me_2_HSi)_3_Si]_2_Ca (**III**) (Chart 1) was along the same lines.^21^ Subsequently, Sekiguchi and co-workers showed that reaction of the dipotassium tetrasilacyclobutadiene dianion K_2_[(^*t*^Bu_2_MeSi)_4_Si_4_] with CaI_2_ led to the formation of an interesting building block calcium tetrakis(di-*tert*-butylmethylsilyl)tetrasilabicyclo[1.1.0]butane-2,4-diide (**IV**) (Chart 1).^22^ More recently, Okuda and co-workers prepared (Ph_3_Si)_2_Ca (**V**) from Ph_3_SiK,^23^ and Mills and Liddle reported the synthesis of THF adducts of (^*t*^Bu_3_Si)_2_Ca and (*t*Bu_2_MeSi)_2_Ca (**VI**) from the respective sodium silanides (Chart 1).^24^

### Silyl Yttrium Compounds

**Chart 2:**
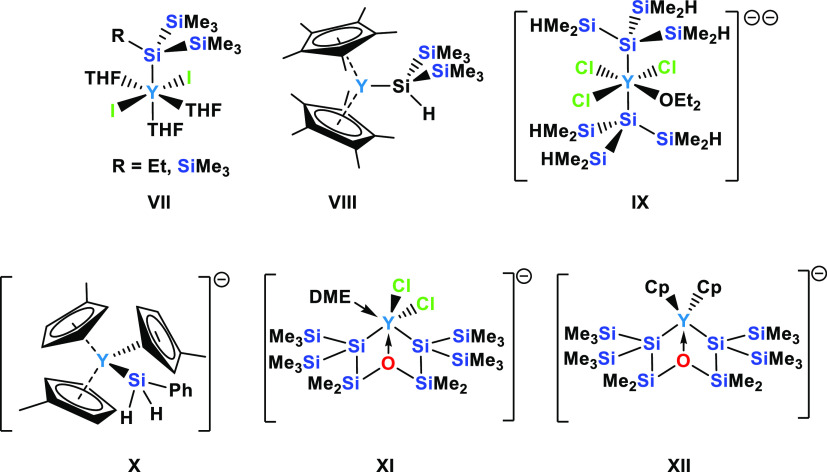
Reported Examples of Yttrium Silanides **VII**,^28^**VIII**,^29^**IX**,^21^**X**,^30^**XI**,^31^ and **XII**(31)

The origin of the organic chemistry of rare-earth metals dates back to the seminal work of Wilkinson and co-workers on metallocenes in the 1950s.^25^ Though with a slow start, this area is nowadays an intensively investigated and flourishing field.^26^ Despite a large number of known organic rare-earth metal compounds (containing metal–carbon bonds), examples of silylated rare-earth complexes are still scarce.^27^ Cases of silylated yttrium compounds are limited to only seven reported examples (Chart 2). These include the neutral compounds R(Me_3_Si)_2_SiYI_2_·(THF)_3_ (R = SiMe_3_, Et) (**VII**) by Sgro and Piers^28^ and Tilley’s Cp*_2_YSiH(SiMe_3_)_2_^29^ (**VIII**) obtained by metathesis of Cp*YCH(SiMe_3_)_2_ with H_2_Si(SiMe_3_)_2_. Sadow and co-workers obtained the dianionic K_2_(Et_2_O)_2_[Y{Si(SiHMe_2_)_3_}_2_Cl_3_(Et_2_O)] (**IX**) from the reaction of (Me_2_HSi)_3_SiK with YCl_3_.^21^ Evans’ ate-complex K[Cp′_3_YSiH_2_Ph]^30^ (**X**) (Cp′ = C_5_H_4_Me) was formed in the reaction of the Y(II) compound K[Cp′_3_Y] with PhSiH_3_. Most recently, our group reported two disilylated yttrium ate-complexes **XI** and **XII** by reaction of a siloxane-containing oligosilanylene diide with YCl_3_ and subsequently with CpNa.^31^

### Silyl Iron Compounds

**Chart 3:**
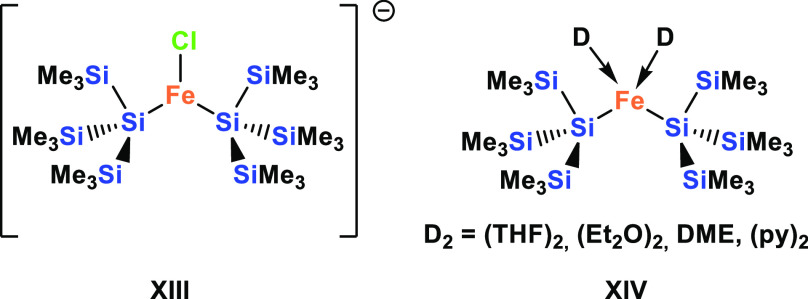
Examples of Fe(II) Complexes with Two Si(Me_3_)_3_ Ligands **XIII**(32,33) and **XIV**(32−34)

While a considerable number of silyl iron complexes is known, examples with two silyl ligands are not so common, and the tris(trimethylsilyl)silyl group is the only oligosilanyl ligand for which disilylated iron complexes are known. Tilley and co-workers reported the reaction of (Me_3_Si)_3_SiLi with FeCl_2_ in DME to give the ferrate complex [Li(DME)_2_]{[(Me_3_Si)_3_Si]_2_FeCl} (**XIII**) (Chart 3).^32,33^ Abstraction of the chloride with Me_3_SiOTf gave the neutral complex [{(Me_3_Si)_3_Si}_2_Fe] (**XIV**), which was obtained as the DME, Et_2_O,^32^ or THF^33^ complex (Chart 3). Only recently, Arata and Sunada demonstrated that the THF adduct of {[(Me_3_Si)_3_Si]_2_Fe} (**XIV**) can be obtained directly from the reaction of (Me_3_Si)_3_SiK with FeBr_2_ in THF, and they further reported its conversion to the dipyridine complex (Chart 3).^34^ With the availability of several oligosilanylene diides, we decided to study the possibility of formation of ferra-cyclosilanes.

## Results and Discussion

### Silyl Calcium Compounds

**Scheme 1:**
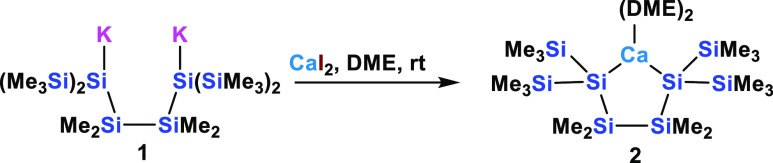
Reaction of Oligosilanylene-1,4-diide **1** with Calcium Diiodide to Calcacyclopentasilane **2**

Dipotassium oligosilanylene-1,4-diide **1**(35,36) is arguably the compound that we used most frequently for the preparation of five-membered heterocyclosilanes. In particular, the compound was reacted with MgBr_2_·Et_2_O to obtain the respective magnesatetrasilacyclopentane,^4^ which may be considered as a reference compound for comparison with other metallacyclosilanes with strong silanide character. Here we treated the dianionic compound **1** with CaI_2_ to obtain the expected calcatetrasilacyclopentane compound **2** with a decent yield of 81% (Scheme 1).

To assess the reactivity of compound **2**, it is convenient to estimate the silanide character by means of ^29^Si NMR spectroscopic analysis. Typically, the silanide character correlates well with the up-field shift of the anionic silicon atom. The chemical shifts of the attached SiMe_3_ groups are also indicative and compared to a neutral compound, typically a down-field shift of the SiMe_3_ signal can be observed. The ^29^Si NMR spectral properties of the dipotassium oligosilanylene-1,4-diide compound **1** are −4.1 (SiMe_3_), −26.7 (SiMe_2_), and −191.6 (SiK) ppm. The values for the respective magnesacyclopentasilane obtained from **1** and MgBr_2_ are −5.4 (SiMe_3_), −27.8 (SiMe_2_), and −176.6 (SiMg) ppm.^4^ Formal replacement of Mg with Zn decreases the silanide character further [−6.3 (SiMe_3_), −26.6 (SiMe_2_), and −152.1 (SiZn) ppm] (Table 1).^37^ In the context of these numbers, the ^29^Si NMR spectrum of **2** [−5.1 (SiMe_3_), −28.4 (SiMe_2_), and −188.0 (SiCa) ppm] suggests the compound to be very ionic with the chemical shift of the metalated silicon atoms being very close to that of the starting material (Table 1). While this value is almost 16 ppm up-field-shifted compared to the bis[tris(trimethylsilyl)silyl]calcium compound **II** reported by Teng and Ruhlandt-Senge (−172.3 ppm),^20^ a similar but less pronounced behavior was observed for the comparison of the respective magnesacyclopentasilane (−176.6 ppm)^4^ and the bis[tris(trimethylsilyl)silyl]magnesium compound (−171.9 ppm).^4,38^

**Table 1:**
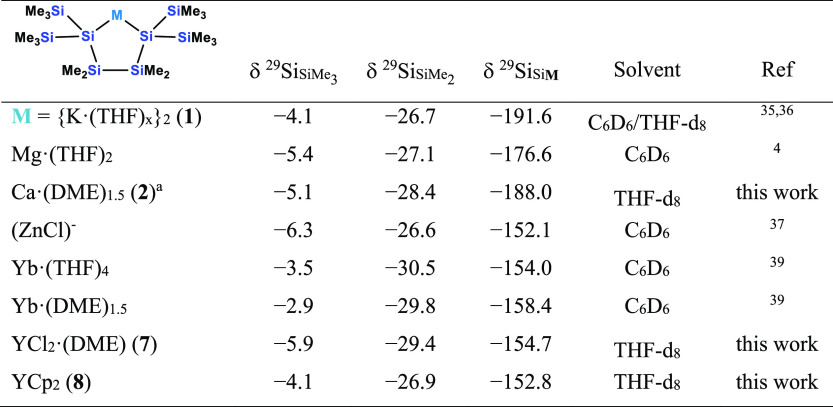
^29^Si NMR Data of 1-Metalla-2,2,5,5-tetrakis(trimethylsilyl)-3,3,4,4-tetramethylcyclopentasilanes

a The DME content of **2** in this case was determined by ^1^H NMR spectroscopy as 1.5. This is different from the number of 2 DME molecules observed in the XRD analysis.

**Figure 1:**
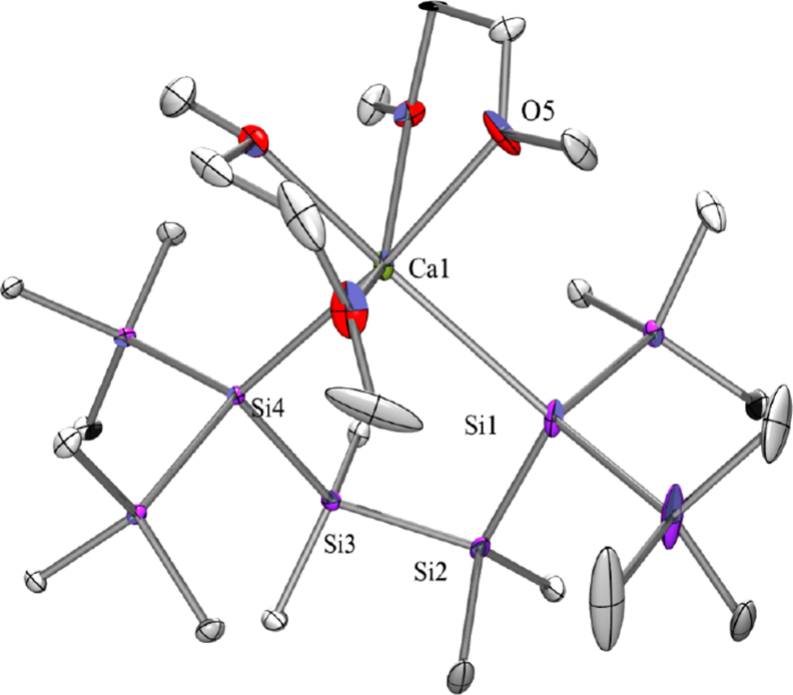
Molecular structure of **2** (thermal ellipsoid plot drawn at the 30% probability level). One co-crystallized DME molecule and all hydrogen atoms are omitted for clarity (bond lengths in Å and angles in deg). Ca(1)–O(4) 2.451(3), Ca(1)–Si(4) 3.0356(13), Ca(1)–Si(1) 3.0534(15), Si(1)–Si(2) 2.358(2), Si(2)–C(20) 1.906(5), Si(2)–Si(3) 2.3648(17), Si(3)–Si(4) 2.3529(15), Si(4)–Si(5) 2.3500(15), O(5)–Ca(1)–Si(4) 171.64(9), O(5)–Ca(1)–Si(1) 91.44(13), Si(4)–Ca(1)–Si(1) 93.13(4), Si(2)–Si(1)–Ca(1) 106.02(5), Si(1)–Si(2)–Si(3) 113.46(6), Si(4)–Si(3)–Si(2) 113.47(6), Si(5)–Si(4)–Ca(1) 128.22(5), Si(3)–Si(4)–Ca(1) 104.79(5).

Single-crystal XRD analysis of **2** (Figure 1) shows the compound to be a regular calcatetrasilacyclopentane with the calcium being further coordinated by two dimethoxyethane (DME) molecules. The five-membered ring is almost flat with all ring atoms except for one of the SiMe_2_ units being co-planar. Endo- and exocyclic Si–Si bonds of the ring are fairly short [2.334(2) to 2.365(2) Å] indicating not much steric strain. Accordingly, the Si–Ca distances of **2** [3.0356(13) and 3.0534(15) Å] are also shorter than those of [(Me_3_Si)_3_Si]_2_Ca·(THF)_3_ (**II**) [3.0422(9) and 3.0862(9) Å].^20^ Due to the cyclic nature of **2** and the long Si–Ca distances, the Si–Ca–Si angle of 93.13(4)° is much smaller than the 125.53° found for **II**.^20^ Several of the known acyclic disilylated calcium compounds feature a linear Si–Ca–Si coordination motif. This was observed for Sadow’s tetra-pyridine adduct of [(HMe_2_Si)_3_Si]_2_Ca (**III**) [Si–Ca: 3.147(3)/3.125(3) Å],^21^ and Okuda’s (Ph_3_Si)_2_Ca·(THF)_4_ (**V**)(3.1503 Å)^23^ and (Ph_3_Si)_2_Ca·(κ^4^-triglyme)(THF) [3.175(3) and 3.242(3) Å].^23^ A non-linear coordination behavior of (Ph_3_Si)_2_Ca was imposed by the use of the macrocyclic tetramine ligand Me_4_TACD [(Ph_3_Si)_2_Ca·(Me_4_TACD): 3.1654(15) Å].^40^

The Ca–O distances of **2** between the calcium ion and the two coordinating DME molecules are longer for the cases where the oxygen atoms are located trans to a silicon atom [2.452(4) and 2.413(4) Å versus 2.398(3) and 2.382(4) Å for the O–Ca–O distances].

**Scheme 2:**
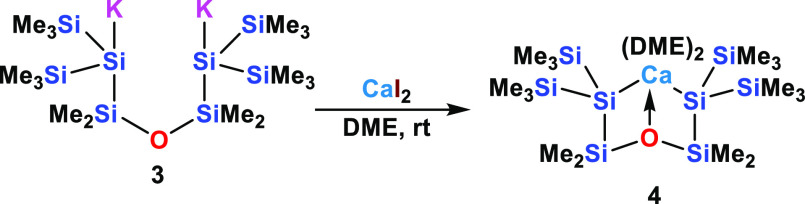
Formation of 1-Calca-4-oxacyclohexasilane **4** from the Reaction of Siloxane Containing Oligosilanylene-1,5-diide **3** with Calcium Diiodide

In order to extend this chemistry, we repeated the reaction of CaI_2_ with the siloxane-containing oligosilanylene-1,5-diide **3**(41) to obtain the disilanyl calcium compound **4** (Scheme 2). Initially, we developed dianion **3** to obtain bidentate silyl ligands with an additional Lewis basic site.^41^ It was used for the synthesis of a number of disilylated Yb(II), Eu(II), and Sm(II) complexes,^41^ and more recently for a number of Ln(III) complexes.^31^

NMR spectroscopic analysis of compound **4** shows it to be fairly similar to **2**. Again the ^29^Si NMR shift of the metallated Si atom of **4** (−179.9 ppm) is close to that of the starting material **3** (−185.7 ppm),^41^ indicating a strong silanide character (Table 2), which is significantly more pronounced than in the corresponding magnesium compound (Table 2).^6^ A comparison of Tables 1 and 2 seems to suggest that the siloxane-containing 1,5-oligosilanylene ligand (Table 2) is of diminished silanide character as the chemical shifts are typically shifted down-field compared to the all-silicon 1,4-treasilanylene ligand (Table 1). This is true for the listed metals: potassium, magnesium, calcium, and ytterbium. However, for yttrium, a reversed trend seems to occur. To understand this, it needs to be pointed out that while the chemical silanide shift is a good approximation for the silanide character, the nature of the ligand and the counterion are not the only variables here. In particular, solvent effects on the shift can be substantial. A strongly coordinating solvent can compete with the silanide ligand for the counter-ion and thus increase the silanide character. Therefore, the nature of the coordinating solvent (THF vs. DME) and also the solvent used for the NMR experiment contribute to the silanide shift. For instance, Table 1 shows that the Yb complex with DME displays a stronger up-field shift than the related THF complex. The fact that the YCl_2_ complex of the siloxane ligand (**XI**) (δ = −161.6 ppm, Table 2) displays stronger silanide character than **7** (δ = −154.7 ppm, Table 1) is in part caused by the fact that it was measured in DME, whereas **7** was measured in the less coordinating solvent THF-*d*_8_.^39^

**Table 2:**
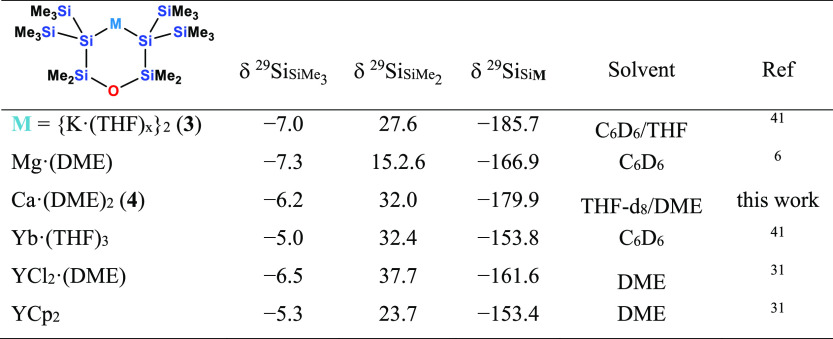
^29^Si NMR Data of 1-Metalla-2,2,6,6-tetrakis(trimethylsilyl)-3,3,5,5-tetramethyl-4-oxacyclohexasilanes

**Figure 2:**
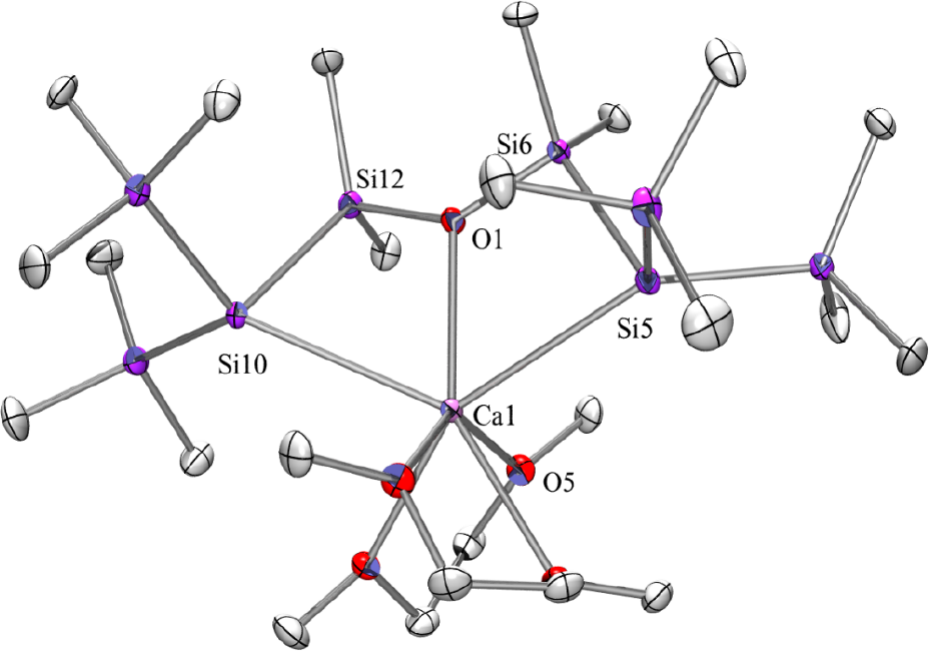
Molecular structure of **4** (thermal ellipsoid plot drawn at the 30% probability level). Of the two molecules in the asymmetric unit, only one is shown. All hydrogen atoms and a co-crystallized pentane molecule are omitted for clarity (bond lengths in Å and angles in deg). Only the anionic part is shown. Ca(1)–O(5) 2.439(2), Ca(1)–O(1) 2.4715(19), Ca(1)–Si(5) 3.1160(10), Ca(1)–Si(10) 3.1492(10), Si(5)–Si(6) 2.3265(11), Si(6)–O(1) 1.6954(19), Si(6)–C(5) 1.867(3), Si(10)–Si(12) 2.3141(11), Si(12)–O(1) 1.700(2), Si(5)–Ca(1)–Si(10) 127.01(3), Si(5)–Ca(1)–Si(12) 95.68(3), Si(10)–Ca(1)–Si(12) 41.29(2), O(1)–Si(6)–Si(5) 101.01(7), Si(12)–Si(10)–Ca(1) 74.80(3), O(1)–Si(12)–Si(10) 100.72(7), Si(10)–Si(12)–Ca(1) 63.90(3), and Si(6)–O(1)–Si(12) 139.69(12).

Single-crystal XRD analysis of **4** (Figure 2) shows significantly elongated Ca–Si interactions of 3.1160(10) and 3.1492(10) Å compared to **2** [3.0356(13) and 3.0534(15) Å] and [(Me_3_Si)_3_Si]_2_Ca·(THF)_3_ (**II**) [3.0422(9) and 3.0862(9) Å].^20^ While the Si–Si distances of **4** are even shorter than those in **2** [ranging from 2.314(1) to 2.341(1) Å], the elongated Si–Ca distances are likely caused by a change from hexa- to hepta-coordination of the calcium ion. This occurs by the additional coordination of the siloxane oxygen atom. The phenomenon of elongated Ca–O bonds in trans-position to Si–Ca, which was mentioned above for **2**, is even more pronounced in **4**, where the Ca–O distances without a *trans*-Si atom amount to values between 2.39 and 2.44 Å, whereas the respective distances in trans-position to a silanide were found to be between 2.519(2) and 2.580(2) Å. The transannular Ca–O interactions of 2.459(2) and 2.481(2) Å are in between these two extremes.

**Scheme 3:**
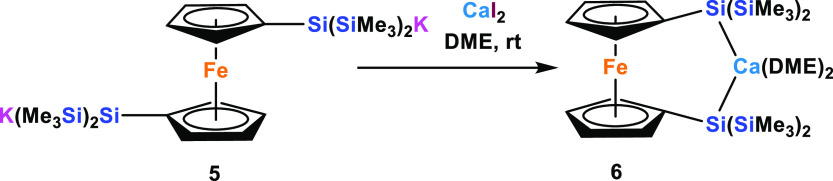
Synthesis of 2-Calca-1,3-disila[3]ferrocenophane **6**

Finally, we were interested in converting the ferrocene-based oligosilanyl dianion **5**(42) to the respective [3]-ferrocenophane calcium compound **6** (Scheme 3). The reaction proceeds again smoothly and ^29^Si NMR analysis of **6** reiterates the trend previously observed for compounds **2** and **4**. Again, the strong silanide character of **6** is reflected by a chemical shift (−116.7 ppm), close to that of the starting material **5** (−121.1 ppm) and shifted to a higher field than that of the corresponding magnesium (−108.5 ppm) and zinc (−92.2 ppm) compounds^42^ (Table 3).

**Table 3:**
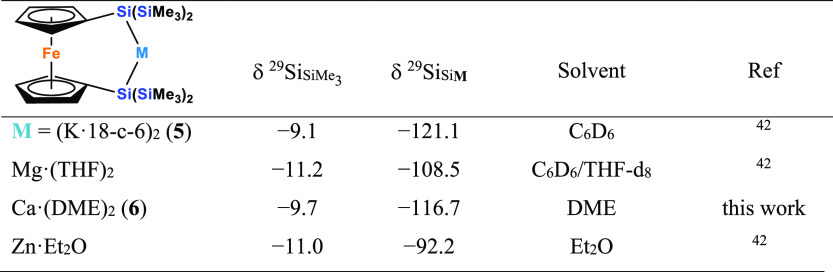
^29^Si NMR Data of [3]Ferrocenophanes with 2-Metalla-1,3-disila Bridges

**Figure 3:**
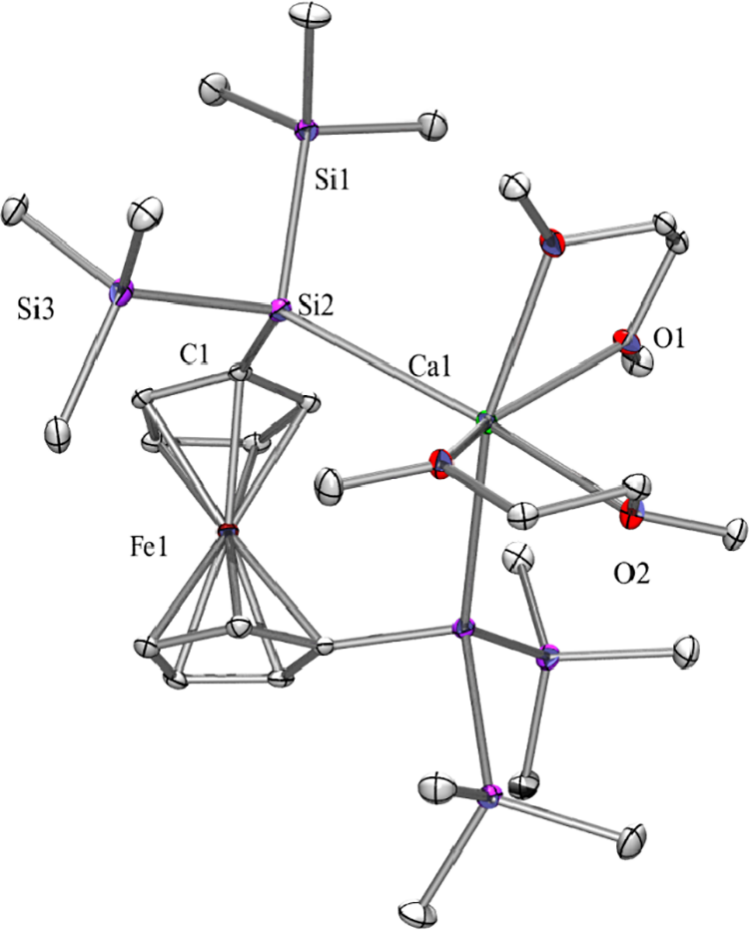
Molecular structure of **6** (thermal ellipsoid plot drawn at the 30% probability level). All hydrogen atoms are omitted for clarity (bond lengths in Å and angles in deg). Ca(1)–O(1) 2.4078(16), Ca(1)–O(2) 2.4212(17), Ca(1)–Si(2) 3.0765(8), Fe(1)–C(2) 2.024(2), O(1)–C(12) 1.430(3), Si(1)–C(8) 1.878(3), Si(1)–Si(2) 2.3592(10), Si(2)–C(1) 1.915(2), Si(2)–Si(3) 2.3486(10), Si(2)–Ca(1)–Si(2A) 98.26(3), C(1)–Si(2)–Si(3) 105.11(8), Si(3)–Si(2)–Si(1) 102.14(3), C(1)–Si(2)–Ca(1) 108.90(7), Si(3)–Si(2)–Ca(1) 126.72(3), Si(1)–Si(2)–Ca(1) 112.08(3), Si(2)–C(1)–Fe(1) 127.29(11).

Single-crystal XRD analysis of **6** (Figure 3) shows a complex with two donating DME molecules and a hexacoordinate calcium atom similar to what was found for **2**. As a consequence, the Ca–Si distance of 3.0765(8) Å is closer to that of **2** than that of **4**. The Si–Si distances of 2.359(1) and 2.348(1) Å again indicate a non-strained structure. The Si–Ca–Si angle of 98.26(3)° is somewhat larger than what was found for **2**. The picture of elongated Ca–O distances in trans-position to a Si–Ca bond is also evident for **6** [2.4212(17) Å (*O*–*Ca*–Si) vs 2.4078(16) Å for *O*–*Ca*–O] but less pronounced.

### Silyl Yttrium Compounds

Our recently reported reaction of the siloxane-containing oligosilanylene dianion **3** with YCl_3_ to the respective disilylated dichloro yttrate-complex **XI**,^31^ led us to reconsider the reaction of dianion **1** in a similar way. Indeed, the reaction of **1** with yttrium trichloride in DME proceeded smoothly, and we obtained dichloroyttratetrasilacyclopentane **7** as an ate-complex with a DME coordinating to the yttrium atom.

^29^Si NMR spectroscopic analysis of **7** showed a doublet signal at −154.7 ppm for the metallated silicon atom with a ^1^*J*_Si–Y_ coupling constant of 56 Hz for the metalated silicon atom (Table 1). For the related product **XI** (see Chart 2),^31^ the respective signal was detected with a chemical shift of −161.6 ppm and a ^1^*J*_Si–Y_ coupling constant of 38 Hz. Although these values are indicating a more ionic character of complex **XI** compared to **7**, some reasons for this somewhat counterintuitive finding are discussed above.

**Figure 4:**
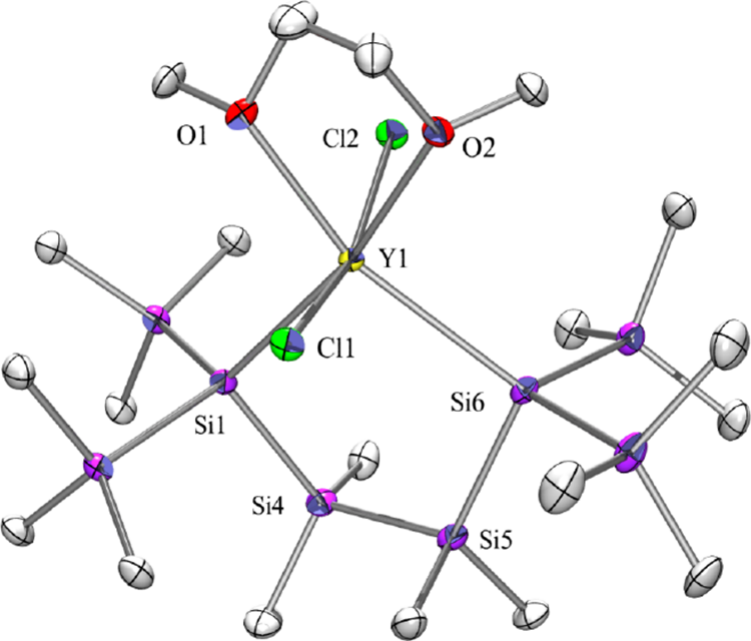
Molecular structure of **7** (thermal ellipsoid plot drawn at the 30% probability level). All hydrogen atoms are omitted for clarity (bond lengths in Å and angles in deg). Only the anionic part is shown. Y(1)–O(2) 2.4215(19), Y(1)–O(1) 2.4455(19), Y(1)–Cl(1) 2.5843(8), Y(1)–Cl(2) 2.5883(8), Y(1)–Si(6) 2.9589(9), Y(1)–Si(1) 2.9704(9), O(1)–C(1) 1.422(4), Si(1)–Si(4) 2.3582(11), Si(2)–C(5) 1.881(3), Si(4)–Si(5) 2.3537(12), Si(5)–Si(6) 2.3513(12), O(2)–Y(1)–O(1) 68.16(7), Cl(1)–Y(1)–Cl(2) 162.33(3), Si(6)–Y(1)–Si(1) 96.66(2), Si(4)–Si(1)–Y(1) 103.74(3), Si(5)–Si(4)–Si(1) 111.30(4), Si(6)–Si(5)–Si(4) 113.35(4).

The solid state structure of **7** (Figure 4), features Si–Y distances of 2.9589(9) and 2.9704(9) Å, about 0.1 Å shorter than those found for **XI** [3.064(2) and 3.057(1) Å].^31^ All other distances, such as Y–Cl and Y–O are very similar for both types of complexes.

It therefore seems valid to assume that the additional Y–O interaction, which distinguishes complex **XI** from complex **7**, is responsible for the increased ionic character of the Si–Y bonds of the former.

**Figure 5:**
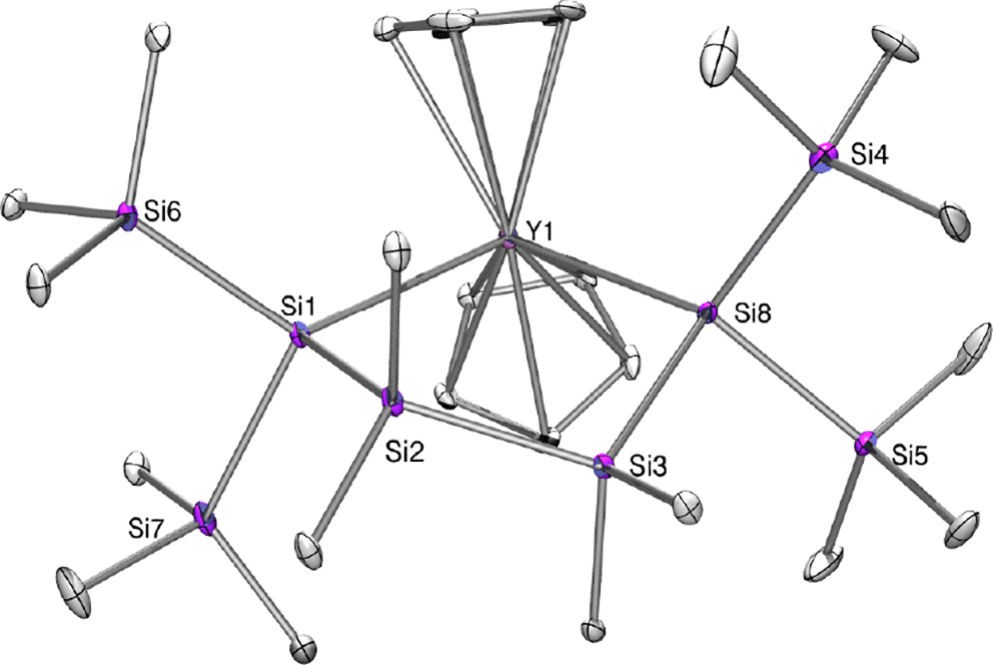
Molecular structure of **8** (thermal ellipsoid plot drawn at the 30% probability level). All hydrogen atoms are omitted for clarity (bond lengths in Å and angles in deg). Only the anionic part is shown. Si(1)–Si(2) 2.359(3), Si(1)–Si(6) 2.366(3), Si(1)–Si(7) 2.425(3), Si(1)–Y(1) 3.006(3), Si(6)–C(36) 1.882(8), Si(3)–Si(2) 2.357(2), Y(1)–C(19) 2.610(5), Y(1)–Si(8) 3.0106(13), Si(2)–Si(1)–Si(6) 100.76(12), Si(2)–Si(1)–Si(7) 106.69(11), Si(6)–Si(1)–Si(7) 101.11(11), Si(2)–Si(1)–Y(1) 100.26(9), Si(6)–Si(1)–Y(1) 127.50(11), Si(8)–Si(3)–Si(2) 112.31(8), Si(3)–Si(2)–Si(1) 113.15(10), Si(1)–Y(1)–Si(8) 97.94(6).

**Scheme 4:**
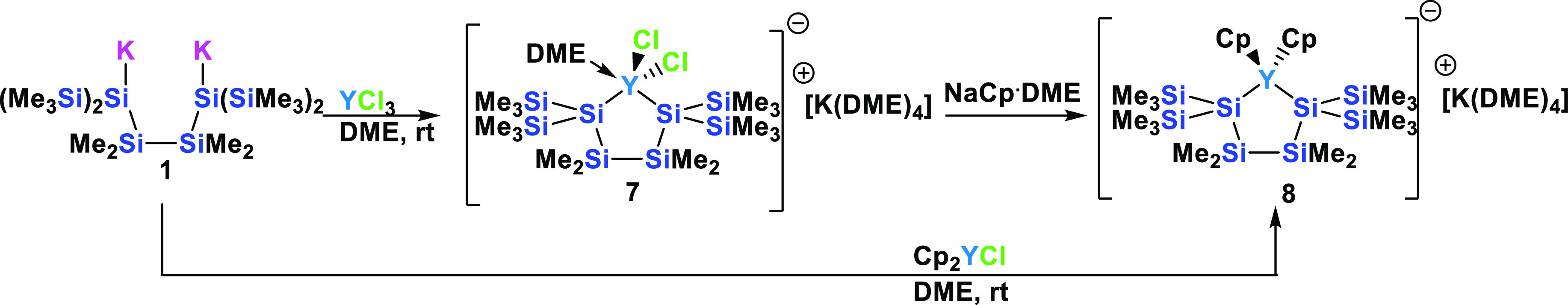
Synthesis of Yttratetrasilacyclopentane Ate-Complexes **7** and **8**

Reaction of complex **7** with two equivalents of NaCp proceeds under substitution of the chlorides against cyclopentadienyl ligands to give the yttracenate complex **8** (Scheme 4). The ^29^Si NMR spectrum of **8,** with a signal at −152.8 ppm (^1^*J*_Si–Y_ = 57 Hz) for the metalated silicon, is fairly similar to that of **7** both with respect to chemical shift and ^1^*J*_Si–Y_ coupling constant (Table 1), suggesting that the degree of covalency of the Si–Y bonds in both compounds is very similar. This is consistent with the fact that the Cp_2_Y ate complex **XII**, features only a small degree of interaction between the Y atom and the siloxane oxygen atom.^31^ While the solid state structure of **8** (Figure 5) features longer Si–Y [3.006(3) and 3.0106(13) Å] bonds than **7** [2.9589(9) and 2.9704(9) Å], these distances are substantially shortened compared to the 3.1315(9) and 3.1459(9) Å observed for complex **XII**.^31^

### Silyl Iron Compounds

As outlined in the introduction, Fe(II) complexes with two silyl groups are not completely uncommon. They are, however, paramagnetic and NMR characterization is not easily possible. Arata and Sunada showed that ^1^H NMR spectra of [(Me_3_Si)_3_Si]_2_Fe(THF)_2_ and [(Me_3_Si)_3_Si]_2_Fe(py)_2_ (**XIV**) can be obtained but feature very broad and strongly shifted lines.^34^

**Scheme 5:**
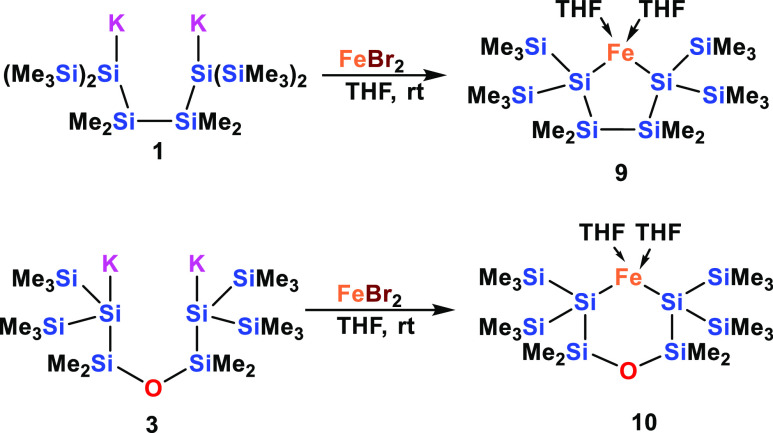
Synthesis of Ferracyclosilanes **9** and **10** by Reactions of the Respective Oligosilanylene Diides **1** and **3** with Iron Dibromide

Our approach to cyclic disilylated Fe(II) complexes follows the same strategy as outlined above for the reactions with CaI_2_ and YCl_3_. Reactions of oligosilanylene diides **1** and **3** with FeBr_2_ thus proceeded to form compounds **9** and **10**, respectively, as purple and ruby-red crystals (Scheme 5).

**Figure 6:**
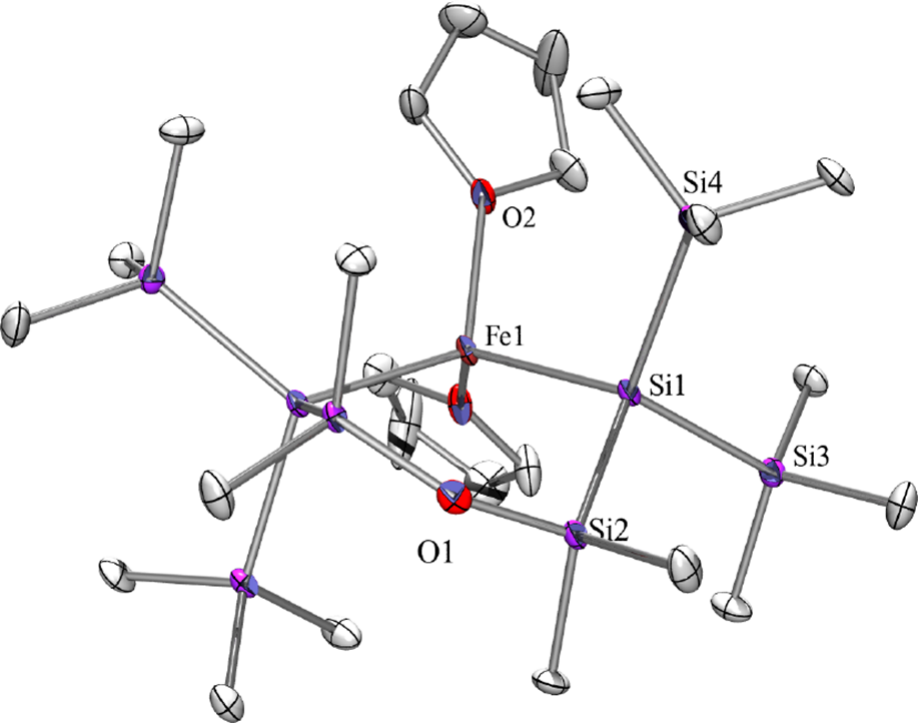
Molecular structure of **10** (thermal ellipsoid plot drawn at the 30% probability level). All hydrogen atoms are omitted for clarity (bond lengths in Å and angles in deg). Fe(1)–O(2) 2.103(4), O(1)–Si(2) 1.653(2), O(2)–C(9) 1.449(8), Si(1)–Si(3) 2.340(2), Si(1)–Si(2) 2.351(2), Si(1)–Si(4) 2.360(2), Si(2)–C(1) 1.869(7), O(2)–Fe(1)–Si(1) 109.39(12), Si(3)–Si(1)–Si(2) 105.75(9), Si(3)–Si(1)–Fe(1) 118.39(8), Si(2)–Si(1)–Fe(1) 105.79(7), O(1)–Si(2)–Si(1) 112.16(19).

The ^1^H NMR spectrum of **9** is similar to that of [(Me_3_Si)_3_Si]_2_Fe(THF)_2_^34^ with the OCH_2_ THF signals shifted to ca. + 25 ppm. Unfortunately, the crystals of **9** were not suitable for single-crystal XRD analysis, but those of **10** proved to be acceptable and structure analysis was possible (Figure 6).

As outlined above, we are aware of only three examples of structurally characterized bis(oligosilanylated) Fe(II) complexes. These are Tilley’s ate-complex Et_4_N{[(Me_3_Si)_3_Si]_2_FeCl} (**XIII**)^32^ and Sunada’s [(Me_3_Si)_3_Si]_2_Fe(py)_2_ and [(Me_3_Si)_3_Si]_2_Fe(THF)_2_ (**XIV**) Chart 3).^34^ These compounds feature Si–Fe bond distances of around 2.50 Å (Table 4) and the structural features of **10** match with those of the Tilley and Sunada compounds very well. Apart from a smaller Si–Fe–Si angle, which is caused by the cyclic nature of the attached silyl groups, the Si–Fe as well as the Si–Si distances are very similar.

**Table 4:**
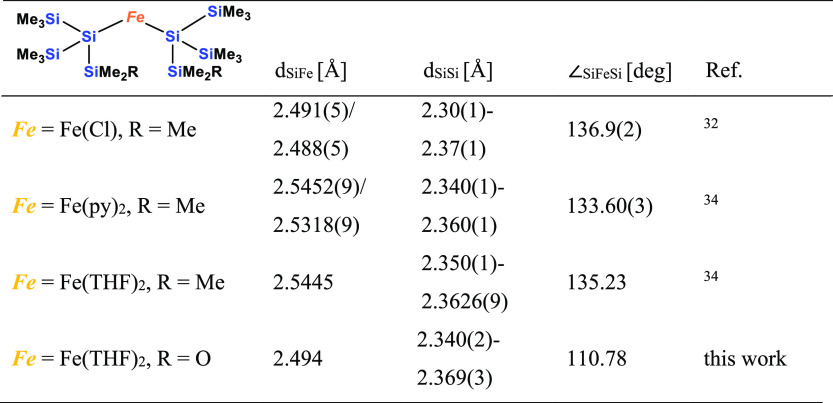
Basic Structural Features of Di(oligosilanylated) Iron(II) Complexes

The conformation of **10** is somewhat unusual. It features a typical large Si–O–Si angle of almost 143°, which we have observed for several other 1-metalla-4-oxa-tetrasilacyclohexanes. This large angle renders the Si–O–Si unit almost as one ring side, thus allowing describing its conformational properties as those of a five-membered ring. While most of the 1-metalla-4-oxa-tetrasilacyclohexanes studied by us so far tend to engage in a distorted envelope conformation with the metal unit as flap,^6,43^ the structure of **10** is more accurately described as a half-chair conformer with the Si–Fe–Si unit and the oxygen atom in a plane and the SiMe_2_ extending below and above the plane (Figure 6).

## Conclusions

In our ongoing pursuit to develop the chemistry of heterocyclosilanes, we used a choice of oligosilanylene diides to prepare heterocyclic silanes with endocyclic Ca, Y, and Fe atoms. Calcacyclosilanes were obtained by reaction of oligosilanylene dianions with CaI_2_. ^29^Si NMR spectroscopic analysis of the compounds indicates a retained strong silanide character, which is markedly more pronounced than in the analogous magnesacyclosilanes and almost resembles that of the potassium silanides used as starting materials. A degree of reactivity located between potassium and magnesium silanides would render silyl calcium compounds interesting silyl anionic reagents.

The reaction of a dipotassium 1,4-oligosilanylene diide with YCl_3_ gave a five-membered yttracyclosilane as an ate-complex with two chlorides still attached to the yttrium atom. Treatment of this compound with two equivalents of NaCp led to a five-membered yttracyclosilane ate-complex containing an yttracene fragment.

NMR spectroscopic analysis of experiments, subjecting the obtained calcacyclosilanes **2** and **4** to reactions with YCl_3_ showed the ^29^Si signatures of **7** and **XI**, respectively. However, these reactions were less clean than those utilizing the potassium silanides **1** and **3**, displaying a small amount of products with Si–Si bonds between the previously anionic silicon atoms. In addition, it was not possible to isolate the crystalline material of the ate-complexes, which should contain either Ca^2+^ or CaCl^+^ counterions.

In calcium and yttrium containing six-membered rings featuring a 3-oxa-tetrasilapentylene fragment, the respective siloxane oxygen atom acts as an additional Lewis basic coordination site for transannular interactions with Ca and Y. When the same ligand fragment was used to prepare a 1-ferra-4-oxatetrasilacyclohexane from 1,4-dipotassio-1,1,4,4-tetrakis(trimethylsilyl)tetramethyltetrasilane and FeBr_2_, no transannular interaction between the iron and oxygen atoms could be observed.

## Experimental Section

### General Remarks

All reactions involving air-sensitive compounds were carried out under an atmosphere of dry nitrogen or argon using either Schlenk techniques or a glove box. All solvents were dried using a column-based solvent purification system.^44^ 2,2,5,5-Tetrakis(trimethylsilyl)decamethylhexasilane,^45^ 1,4-dipotassio-1,1,4,4-tetrakis(trimethylsilyl)tetramethyltetrasilane (**1**),^36^ 1,3-bis[potassiobis(trimethylsilyl)silyl]-1,1,3,3-tetramethyldisiloxane (**3**),^41^ Cp_2_YCl,^46^ 1,1′-bis[tris(trimethylsilyl)silyl]ferrocene,^42^ and **5**(42) were prepared following published procedures. All other chemicals were obtained from different suppliers and used without further purification.

^1^H (300 MHz), ^13^C (75.4 MHz), and ^29^Si (59.3 MHz) NMR spectra were recorded on a Varian INOVA 300 spectrometer. If not noted otherwise all samples were measured in C_6_D_6_. To compensate for the low isotopic abundance of ^29^Si, the INEPT pulse sequence was used for the amplification of the signal.^47−49^ Spectra are calibrated to the deuterium resonance of the solvent (C_6_D_6_)^50^ and referenced to tetramethysilane (TMS).^51^

Elementary analyses were carried out using a Heraeus VARIO ELEMENTAR.

### X-ray Structure Determination

For X-ray structure analyses, crystals were mounted onto the tip of glass fibers, and data collection was performed with a BRUKER-AXS SMART APEX CCD diffractometer using graphite-monochromated Mo Kα radiation (0.71073 Å). The data were reduced to *F*_o_^2^ and corrected for absorption effects with SAINT^52^ and SADABS,^53,54^ respectively. The structures were solved by direct methods (SHELXT)^55^ and refined by the full-matrix least-squares method (SHELXL),^56^ and in some cases, OLEX2^57^ was used. If not noted otherwise, all non-hydrogen atoms were refined with anisotropic displacement parameters. All hydrogen atoms were located in calculated positions to correspond to standard bond lengths and angles. All diagrams are drawn with 30% probability thermal ellipsoids and all hydrogen atoms are omitted for clarity. Crystallographic data for the structures of compounds **2**, **4**, **6**, **7**, **8**, and **10** reported in this paper are deposited with the Cambridge Crystallographic Data Center as supplementary publication no. CCDC-2071455 (**2**), 2071456 (**4**), 2071458 (**6**), 2071463 (**7**), 2071461 (**8**), and 2071464 (**10**). Copies of data can be obtained free of charge at: http://www.ccdc.cam.ac.uk/products/csd/request/. Figures of solid-state molecular structures were generated using Ortep-3 as implemented in WINGX^58^ and rendered using POV-Ray 3.6.^59^

### 1-Calca-2,2,5,5-tetrakis(trimethylsilyl)tetramethylcyclopentasilane·(DME)_1.5_ (**2**)

A solution of 2,2,5,5-tetrakis(trimethylsilyl)decamethylhexasilane (101 mg, 0.17 mmol) and potassium *tert*-butoxide (39 mg, 0.35 mmol) in THF (2 mL) was stirred at rt for 19 h. Full conversion to disilanide **1** was confirmed by ^29^Si NMR. Volatiles were removed under reduced pressure, the residue dissolved in DME (1.5 mL) and the resulting bright yellow solution was added dropwise within 2 min to a slurry of calcium diiodide (52 mg, 0.18 mmol) in DME (1 mL). The mixture was stirred for 2 h before the precipitated potassium iodide was removed by filtration. Colorless crystals of **2** (180 mg, 81%) were obtained from a toluene/DME solution. NMR (δ in ppm, *d*_8_-THF) ^1^H: 3.48 (s, 6H), 3.31 (s, 9H), 0.20 (s, 12H), 0.11 (s, 36H). ^13^C: 72.7, 59.2, 7.7, 1.9. ^29^Si: −5.1 (s, SiMe_3_), −28.4 (s, SiMe_2_), −188.0 (s, Si_*q*_).

### 1-Calca-4-oxa-2,2,6,6-tetrakis(trimethylsilyl)tetramethylcyclohexasilane (**4**)

1,3-Bis[potassiobis(trimethylsilyl)silyl]-1,1,3,3-tetramethyldisiloxane (**3**) was prepared starting from tetrakis(trimethylsilyl)silyl-1,1,3,3-tetramethyldisiloxane (104 mg, 0.17 mmol) and potassium *tert*-butoxide (39 mg, 0.35 mmol) in THF (2 mL). After 19 h at rt, full conversion was detected by ^29^Si NMR spectroscopy. Volatiles were removed under reduced pressure, DME (1.5 mL) was added and the bright yellow solution was added dropwise within 2 min to a slurry of calcium diiodide (52 mg, 0.18 mmol) in DME (1 mL). The mixture was stirred for 2 h before the precipitate was removed through filtration. Colorless crystals of **4** (460 mg, 88%) were obtained from a pentane/DME solution. NMR (δ in ppm, *d*_8_-THF) ^1^H: 3.49 (s, 8H), 3.37 (s, 12H), 0.38 (s, 12H), 0.12 (s, 36H). ^13^C: 72.7, 59.6, 10.2, 6.6. ^29^Si: 32.0 (s, SiMe_2_), −6.2 (s, SiMe_3_), −178.9 (s, Si_*q*_).

### 1,1′-*ansa*-[2,2,4,4-Tetramethyl-1,1,5,5-tetrakis(trimethylsilyl)-3-calca(^•^2DME)-pentasilan-1,5-ylene]ferrocene (**6**)

Compound **5** (freshly prepared from 1,1′-bis[tris(trimethylsilyl)silyl]ferrocene (86 mg, 0.13 mmol) and KO^*t*^Bu (30 mg, 0.27 mmol)) in DME (2 mL) was added dropwise within 2 min to neat calcium diiodide (42 mg, 0.14 mmol). The resulting orange suspension was stirred for 3 h at rt before full conversion was detected by NMR analysis. Orange crystals of **6** (90 mg, 95%) were obtained from this solution at −50 °C. mp 206 °C (decomp). NMR (δ in ppm, D_2_O-capillary/DME) ^1^H: 3.94 (t, *J* = 1.5 Hz, 4H), 3.55 (t, *J* = 1.5 Hz, 4H), 0.05 (s, 36H). ^13^C: 78.2, 76.3, 68.0, 3.9. ^29^Si: −9.7 (s, SiMe_3_), −116.7 (s, Si_*q*_).

### Potassium·4·DME 1-Yttra-2,2,5,5-tetrakis(trimethylsilyl)tetramethylcyclopenta-silane-1-ate DME (**7**)

2,2,5,5-Tetrakis(trimethylsilyl)decamethylhexasilane (150 mg, 0.25 mmol) and KO^*t*^Bu (58 mg, 0.52 mmol) were dissolved in DME (3 mL) and left for reaction at rt for 24 h after which NMR-analysis showed full conversion toward **1**. Volatiles were removed under reduced pressure and the residue was dissolved in DME (1 mL). This solution was added dropwise to a slurry of yttrium trichloride (53 mg, 0.27 mmol) in DME (1 mL) and stirred at rt for 4.5 h. The resulting cloudy, slightly yellow reaction mixture was subjected to NMR-analysis, showing full conversion. Precipitates were removed through centrifugation and filtration. Colorless crystals of **7** (178 mg, 64%) were obtained from a pentane/DME solution at −50 °C. NMR (δ in ppm, *d*_8_-THF) ^1^H: 0.23 (s, 12H), 0.16 (s, 36H). ^13^C: 6.7, 1.7. ^29^Si: −5.9 (s, SiMe_3_), −29.4 (s, SiMe_2_), −154.7 (d, ^1^*J*_Si–Y_ = 56 Hz, Si_*q*_).

### Potassium·4·DME 1,1-Bis(cyclopentadienyl)-1-yttra-2,2,5,5-tetrakis(trimethylsilyl)-tetramethylcyclopenta-silane-1-ate (**8**)

#### Method A

A solution of **1** [obtained from 2,2,5,5-tetrakis(trimethylsilyl)decamethylhexasilane (156 mg, 0.26 mmol) and KO^*t*^Bu (59 mg, 0.53 mmol)] in DME (1 mL) was added dropwise to a slurry of bis(cyclopentadienyl)yttriumchloride (64 mg, 0.25 mmol) in DME (1 mL) and stirred at rt for 4.5 h. The resulting cloudy, bright yellow reaction mixture was subjected to NMR-analysis showing full conversion. Precipitates were removed by centrifugation and filtration. Colorless crystals of **8** (206 mg, 76%) were obtained from a pentane/DME solution at −50 °C. NMR (δ in ppm, *d*_8_-THF) ^1^H: 6.24 (d, *J*_HY_^2^ = 0.4 Hz, 10H), 0.21 (s, 36H), 0.18 (s, 12H). ^13^C: 108.9 (*pseudo*-t, *J*_C–Y_ = 3.2 Hz), 72.7 (*pseudo*-t, *J*_C–Y_ = 2 Hz), 59.0 (d, *J*_C–Y_ = 2 Hz), 7.4, 2.1. ^29^Si: −3.6 (s, SiMe_3_), −26.4 (s, SiMe_2_), −152.0 (d, ^1^*J*_Si–Y_ = 57 Hz, Si_*q*_).

#### Method B

To a solution of 7 [using 2,2,5,5-tetrakis(trimethylsilyl)decamethylhexasilane (151 mg, 0.25 mmol), KO^*t*^Bu (57 mg, 0.51 mmol), and YCl_3_ (53 mg, 0.27 mmol)] in DME (1 mL), a solution of sodium cyclopentadienyl·DME (91 mg, 0.511 mmol) was added. The reaction mixture was stirred for 80 min at rt. Insoluble parts were removed through centrifugation and filtration, and pentane (9 mL) was added, leading to a biphasic mixture. The lower ionic phase was separated by decantation and was subjected to NMR-analysis, showing pure **8** (133 mg, 49%).

### 1-Ferra-2,2,5,5-tetrakis(trimethylsilyl)tetramethylcyclopentasilane·(THF)_2_ (**9**)

A solution of 2,2,5,5-tetrakis(trimethylsilyl)decamethylhexasilane (151 mg, 0.25 mmol) and potassium *tert*-butoxide (58 mg, 0.52 mmol) in THF (3 mL) was left to react at rt for 23 h. The resulting orange solution was added dropwise to a slurry of iron dibromide (59 mg, 0.274 mmol) in THF (1 mL) and stirred for 3 h. During the addition, the solution first turned deep purple and then brown. Volatiles were removed under reduced pressure, and pentane (3 mL) was added. Precipitated salts were removed through centrifugation and filtration, and purple crystals of **9** (130 mg, 79%) were obtained from the remaining solution at −50 °C. mp 104–106 °C. NMR (δ in ppm, *d*_8_-THF) ^1^H: 24.90 (s, 8H), 1.26 (s, 8H), 0.94 (s, 12H), 0.26 (s, 36H). Neither ^13^C nor ^29^Si NMR spectra could be obtained due to the paramagnetism of **9**. Anal. Calcd. for C_24_H_64_FeO_2_Si_8_ (665.30): C, 43.33; H, 9.70. Found: C, 42.71; H, 10.01.

### 1-Ferra-4-oxa-2,2,6,6-tetrakis(trimethylsilyl)tetramethylcyclohexasilane·2·THF (**10**)

The compound was prepared following the same procedure as for **9** but using 1,3-bis(tris(trimethylsilyl)silyl)-1,1,3,3-tetramethyldisiloxane (150 mg, 0.24 mmol), potassium *tert*-butoxide (55 mg, 0.49 mmol), and iron dibromide (56 mg, 0.26 mmol) in THF (1 mL). Upon addition, the solution turned bright pink and then dark brown. Volatiles were removed under reduced pressure and pentane (2 × 2 mL) was added. Precipitates were removed through centrifugation and filtration and ruby red crystalline **10** (120 mg, 74%) was obtained from this solution at −50 °C. mp 118 °C (decomp). NMR (δ in ppm) ^1^H: 3.64 (br s, 8H), 1.24 (br s, 8H), 0.33 (2 × br s, 48H). Neither ^13^C nor ^29^Si NMR spectra could be obtained from paramagnetic **10**. Anal. Calcd. for C_24_H_64_FeO_3_Si_8_ (681.30): C, 42.31; H, 9.47. Found: C, 41.69; H, 9.62.

## Supporting Information Available

Crystallographic data of all structural characterized compounds; ^1^H, ^13^C, and ^29^Si NMR spectra for compounds **2**, **4**, **6**, **7**, and **8**; and ^1^H NMR spectra of **9** and **10** (PDF)

## Dedication

Dedicated to Prof. Thomas Müller (Universität Oldenburg), on the occasion of his 60^th^ birthday.
