# Supplementary material for: Metallacyclosilanes of Calcium, Yttrium, and Iron
Source: Inorg Chem. 2022 Oct 25;61(44):17527–36. doi: 10.1021/acs.inorgchem.2c02508 (PMC9644368; doi:10.1021/acs.inorgchem.2c02508)
Supplement: Supplementary file 1 — ic2c02508_si_001.pdf [file ic2c02508_si_001.pdf]

Supporting Information for

Metallacyclosilanes of Calcium, Yttrium and Iron

Alexander Pöcheim, Rainer Zitz, Julia Hoenigsberger, Christoph Marschner,* and Judith Baumgartner*

Institut für Anorganische Chemie, Technische Universität Graz, Stremayrgasse 9, 8010 Graz, Austria

e-mail: jdthbaumgartner@gmail.com, christoph.marschner@tugraz.at

Contents

1. Crystallographic tables of compounds **S2**
2. NMR spectra **S4**
   - ^{1}H, ^{13}C and ^{29}Si Spectra **2** **S4**
   - ^{1}H, ^{13}C and ^{29}Si Spectra **4** **S7**
   - ^{1}H, ^{13}C and ^{29}Si Spectra **6** **S10**
   - ^{1}H, ^{13}C and ^{29}Si Spectra **7** **S13**
   - ^{1}H, ^{13}C and ^{29}Si Spectra **8** **S16**
   - ^{1}H Spectrum **9** **S19**
   - ^{1}H Spectrum **10** **S20**

1. Crystallographic Tables

Table S1. Crystallographic data for compounds **2**, **4**, and **6**.

	**2**	**4**	**6**
Empirical formula	$C_{28}H_{78}O_6CaSi_8$	$C_{53}H_{148}Ca_2O_{10}Si_{16}$	$C_{30}H_{64}CaFeO_4Si_6$
M_w	775.70	1475.31	753.28
Temperature [K]	100(2)	100(2)	100(2)
Size [mm]	0.42×0.20×0.16	0.50×0.32×0.22	0.44×0.38×0.22
Crystal system	monoclinic	monoclinic	orthorhombic
Space group	P2(1)	P2(1)	Pbcn
a [Å]	9.819(2)	14.756(3)	13.354(2)
b [Å]	11.661(2)	18.701(4)	18.411(3)
c [Å]	21.097(4)	16.525(4)	19.425(3)
α [°]	90	90	90
β [°]	99.219(3)	92.062(4)	90
γ [°]	90	90	90
V [Å ^{3}]	2384(2)	4557(2)	4776(2)
Z	2	2	4
ρ_{calc} [gcm^{-3}]	1.080	1.075	1.048
Absorption coefficient [mm^{-1}]	0.364	0.376	0.599
F(000)	852	1620	1624
θ range	1.96<θ<26.37	1.65<θ<26.37	1.88<θ<26.34
Reflections collected/unique	19078/9609	36388/18336	25198/4869
Completeness to θ [%]	99.7	99.8	99.7
Data/restraints/parameters	9609/1/421	18336/1/773	4869/0/199
Goodness of fit on F^2	1.19	1.09	1.11
Final R indices [I>2σ(I)]	R1=0.060, *w*R2=0.130	R1=0.043, *w*R2=0.100	R1=0.045, *w*R2=0.099
R indices (all data)	R1=0.062, *w*R2=0.13	R1=0.045, *w*R2=0.101	R1=0.049, *w*R2=0.103
Largest diff. Peak/hole [e$^-$/ Å^3]	0.83/−0.67	0.58/−0.26	0.51/−0.34

Table S2. Crystallographic data for compounds **7**, **8**, and **10**.

	**7**	**8**	**10**
Empirical formula	$C_{36}H_{98}Cl_2KO_{10}Si_8Y$	$C_{42}H_{98}KO_8Si_8Y$	$C_{24}H_{64}FeO_3Si_8$
M_w	1114.77	1083.93	681.32
Temperature [K]	100(2)	100(2)	100(2)
Size [mm]	0.32×0.28×0.14	0.33×0.30×0.28	0.40×0.34×0.14
Crystal system	triclinic	triclinic	monoclinic
Space group	P-1	P-1	I2/a
a [Å]	11.705(2)	13.385(3)	19.454(5)
b [Å]	17.469(3)	15.407(4)	10.600(3)
c [Å]	17.866(3)	15.813(4)	21.198(5)
α [°]	76.663(3)	97.200(4)	90
β [°]	75.053(3)	90.284(4)	116.35(2)
γ [°]	84.630(3)	103.514(4)	90
V [Å ^{3}]	3432(2)	3144(2)	3917(2)
Z	2	2	4
ρ_{calc} [gcm^{-3}]	1.079	1.145	1.155
Absorption coefficient [mm^{-1}]	1.165	1.185	0.652
F(000)	1196	1168	1480
θ range	$1.21<\theta<26.34$	$1.57<\theta<26.37$	$2.14<\theta<26.30$
Reflections collected/unique	27226/13718	25134/12677	14911/3959
Completeness to θ [%]	97.9	98.6	99.4
Data/restraints/parameters	13718/0/549	12677/0/672	3959/0/172
Goodness of fit on F^2	0.99	1.069	1.42
Final R indices [$I>2\sigma(I)$]	R1=0.043, *w*R2=0.105	R1=0.067, *w*R2=0.167	R1=0.094, *w*R2=0.195
R indices (all data)	R1=0.061, *w*R2=0.111	R1=0.089, *w*R2=0.183	R1=0.095, *w*R2=0.196
Largest diff. Peak/hole [e^-/ $Å^3$]	0.81/−0.39	1.80/−1.13	0.79/−0.88

2. ^{1}H, ^{13}C, and ^{29}Si NMR spectra

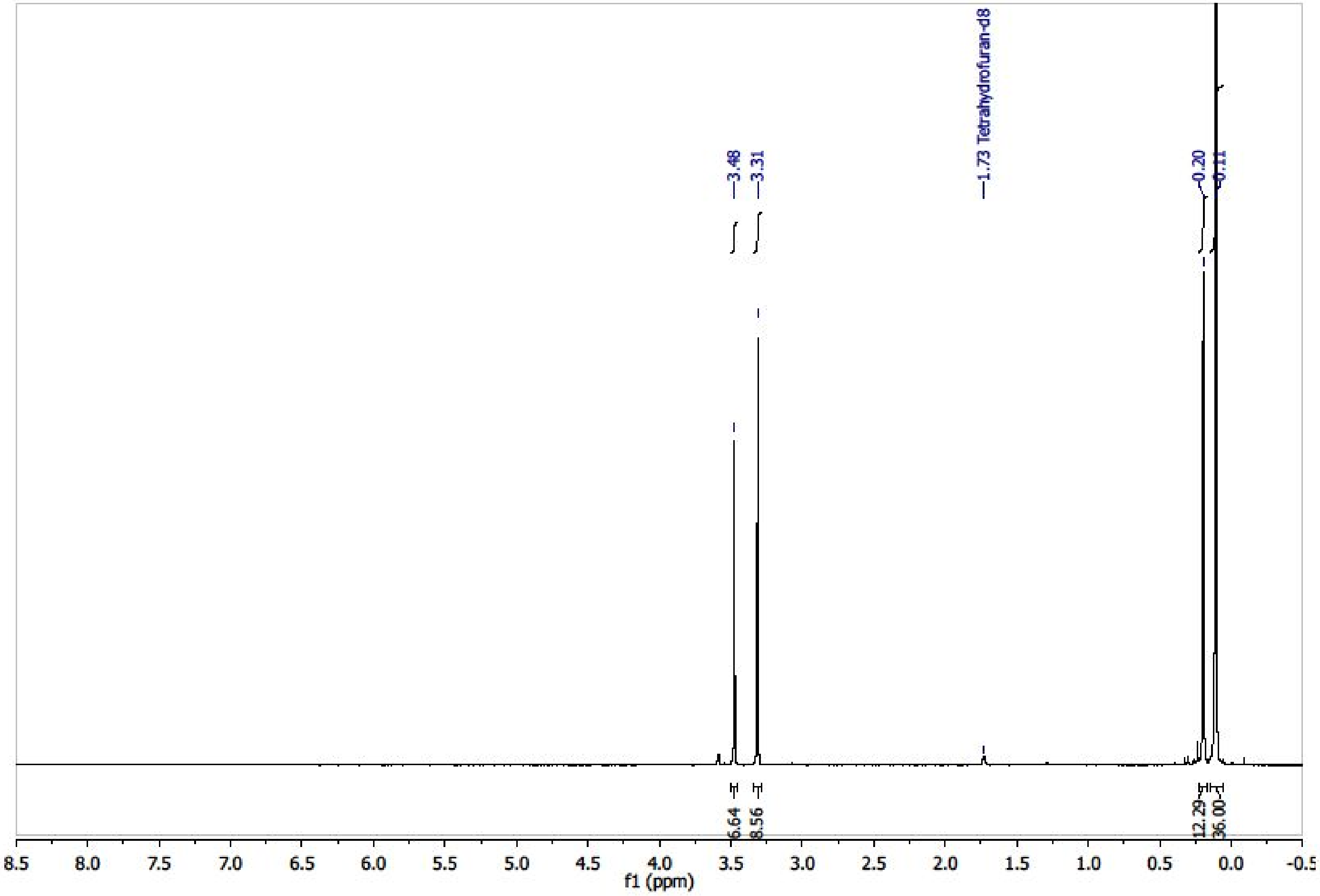

Figure S1. ^{1}H NMR spectrum of compound **2** in THF-d_8

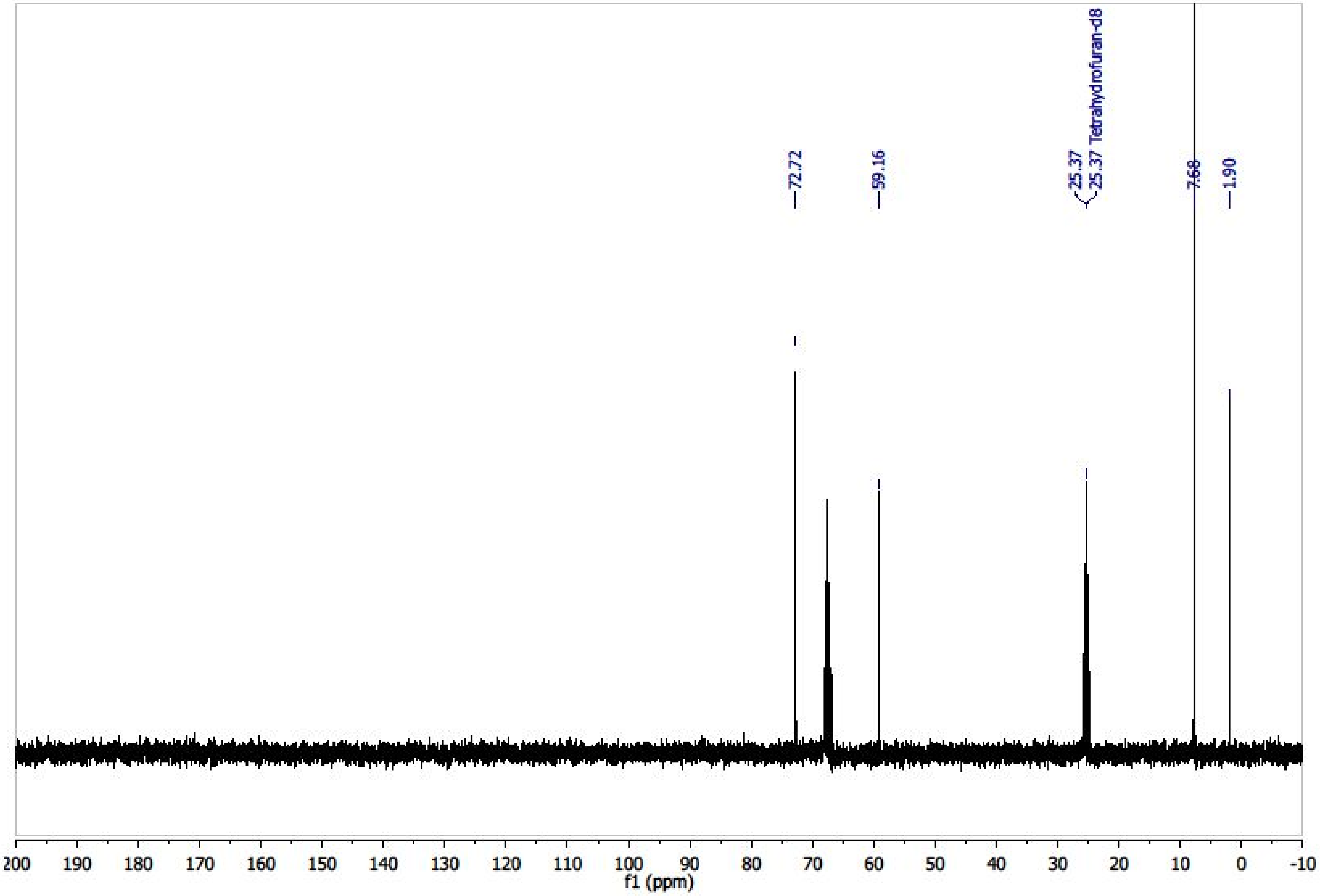

Figure S2. ^{13}C NMR spectrum of compound **2** in THF-d_8

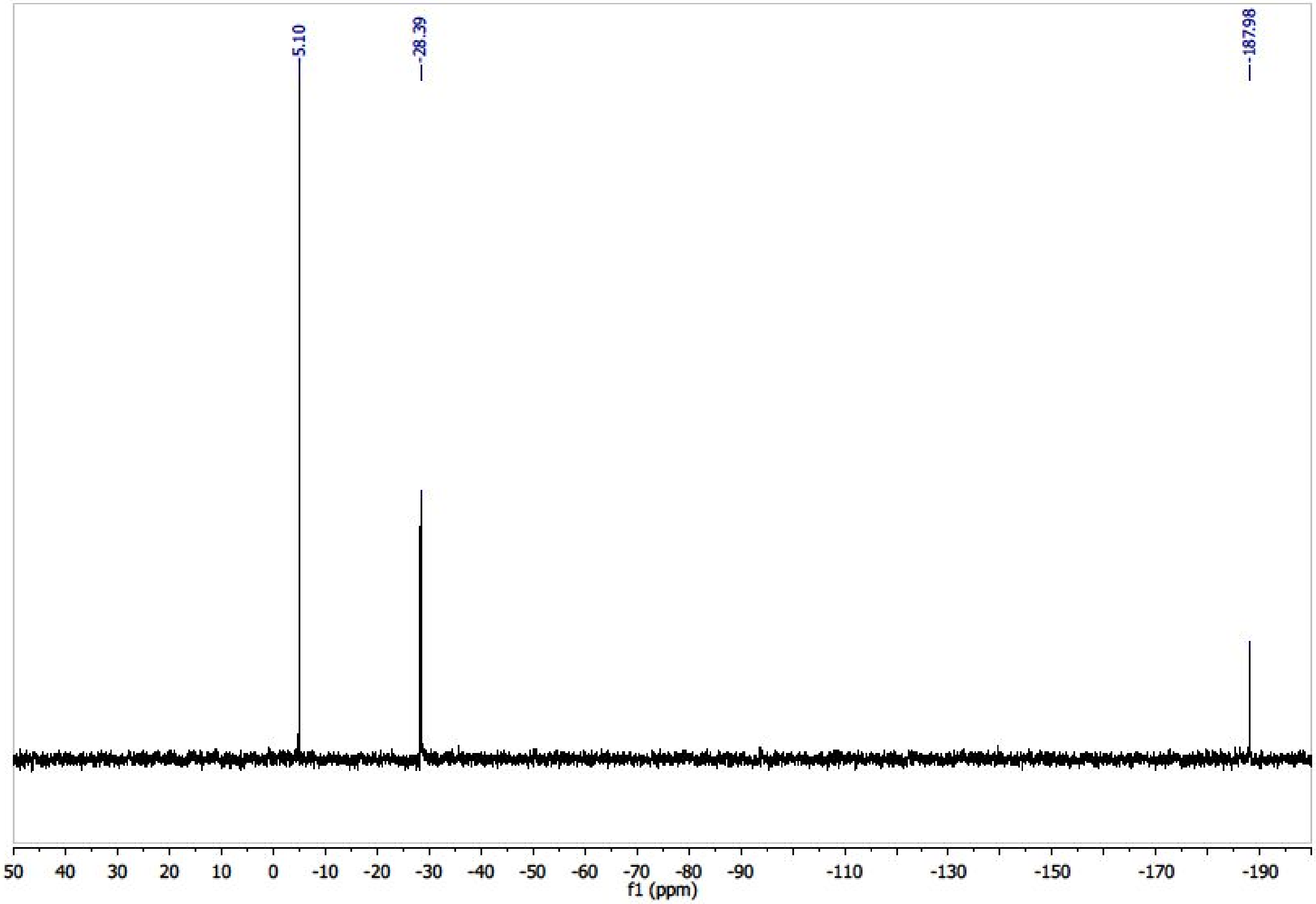

Figure S3. ^{29}Si INEPT NMR spectrum of compound **2** in THF-d_8

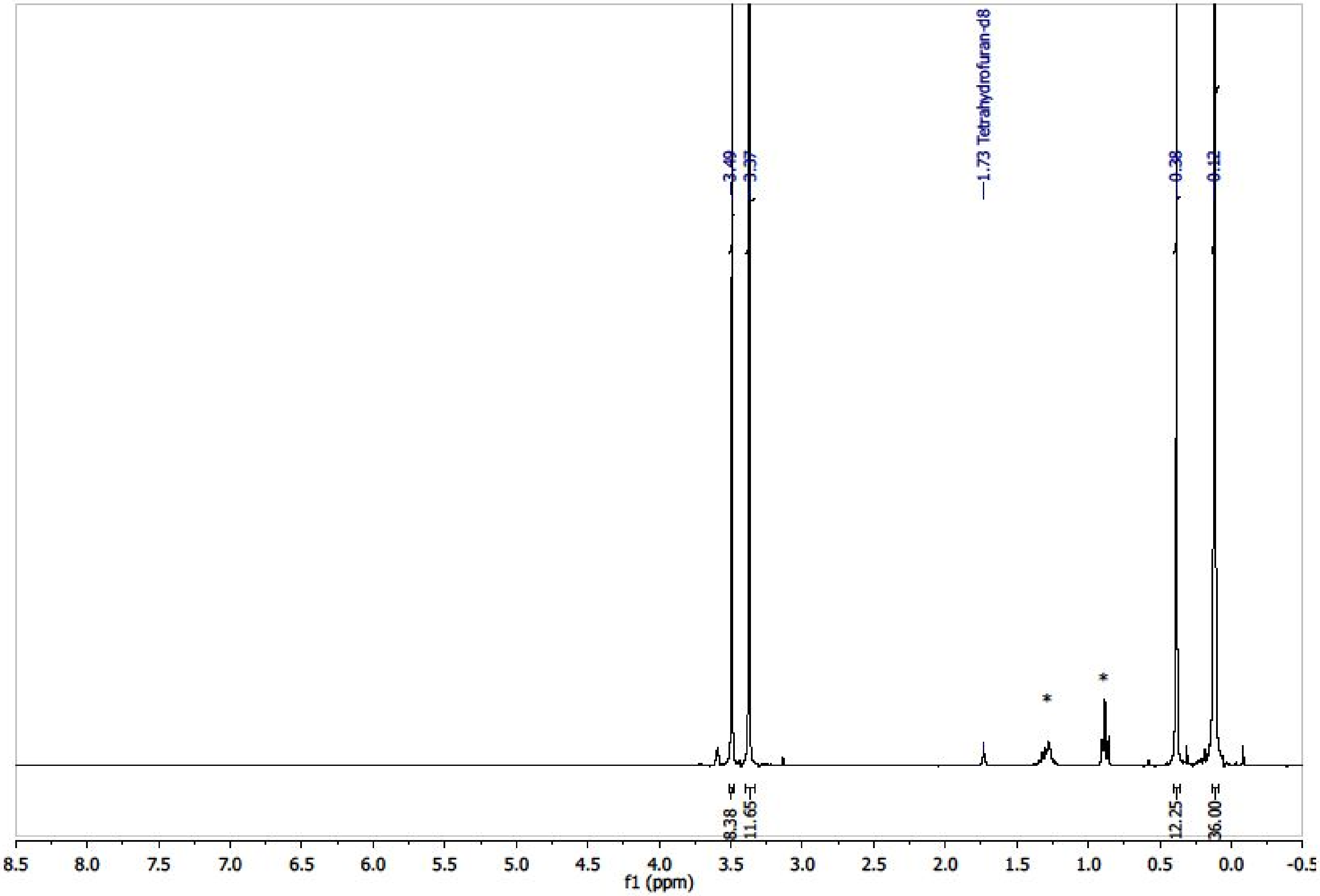

Figure S4. ^{1}H NMR spectrum of compound **4** in THF-d_8 (* = residual pentane)

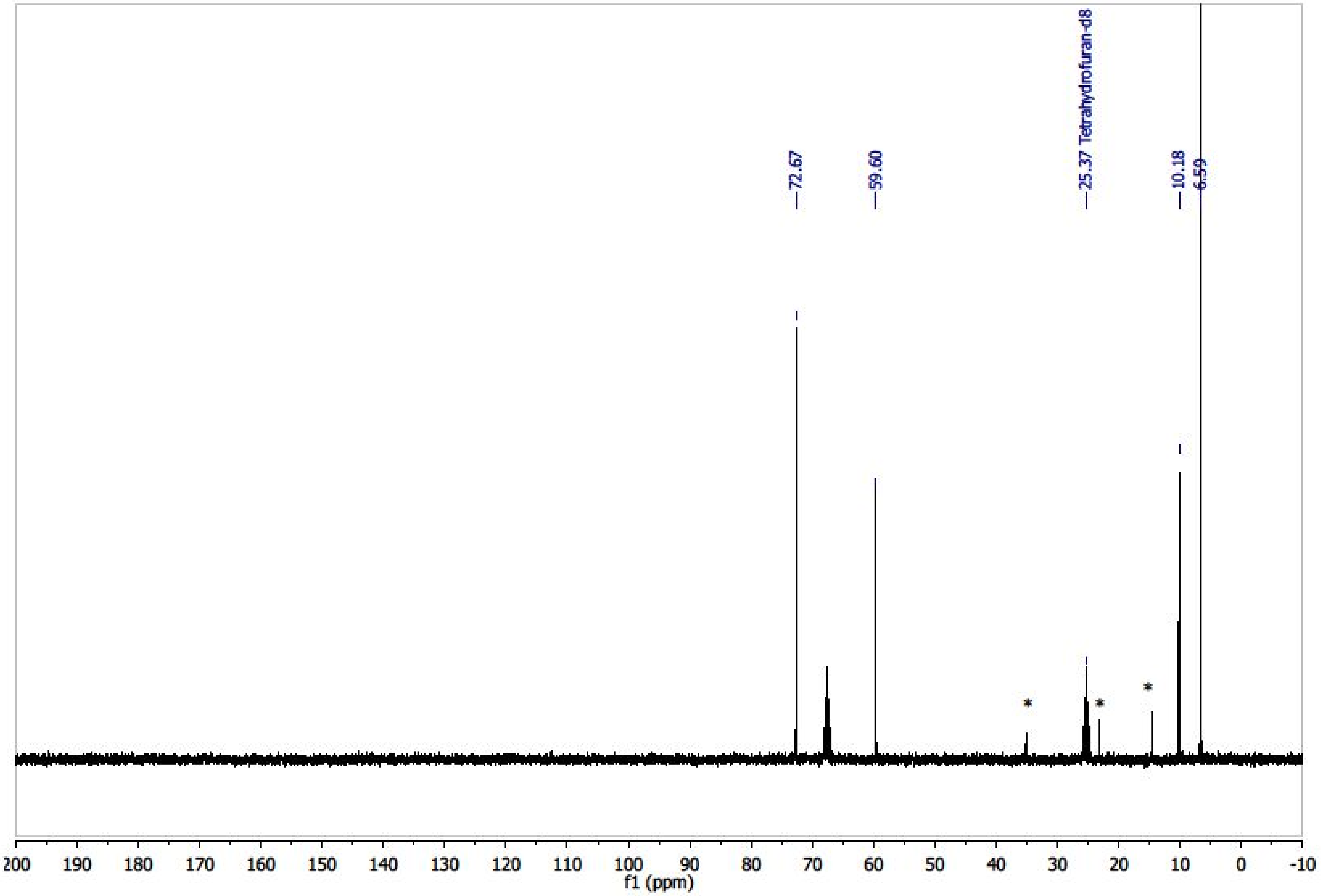

Figure S5. ^{13}C NMR spectrum of compound **4** in THF-d_8 (* = residual pentane)

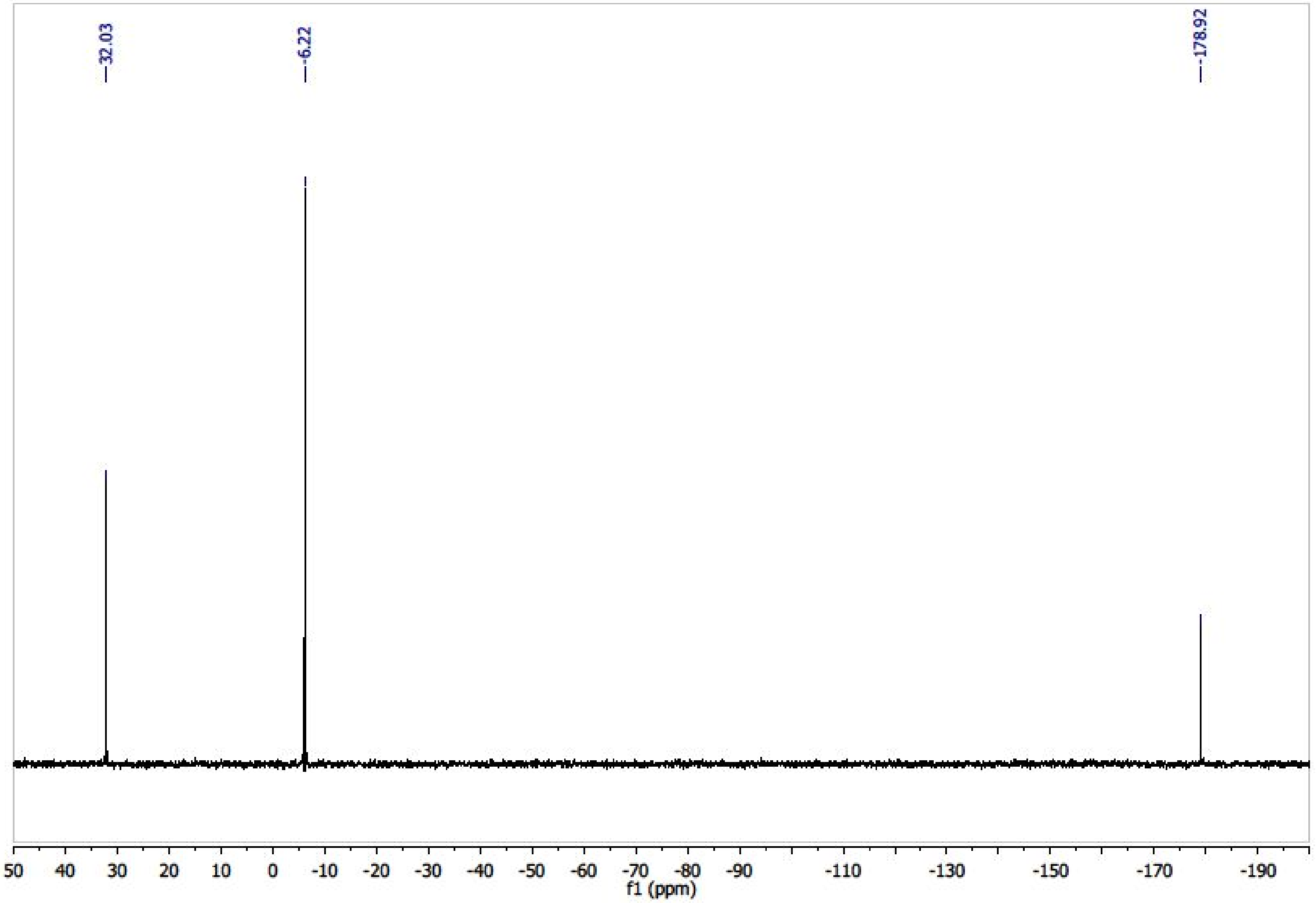

Figure S6. ^{29}Si INEPT NMR spectrum of compound **4** in THF-d_8

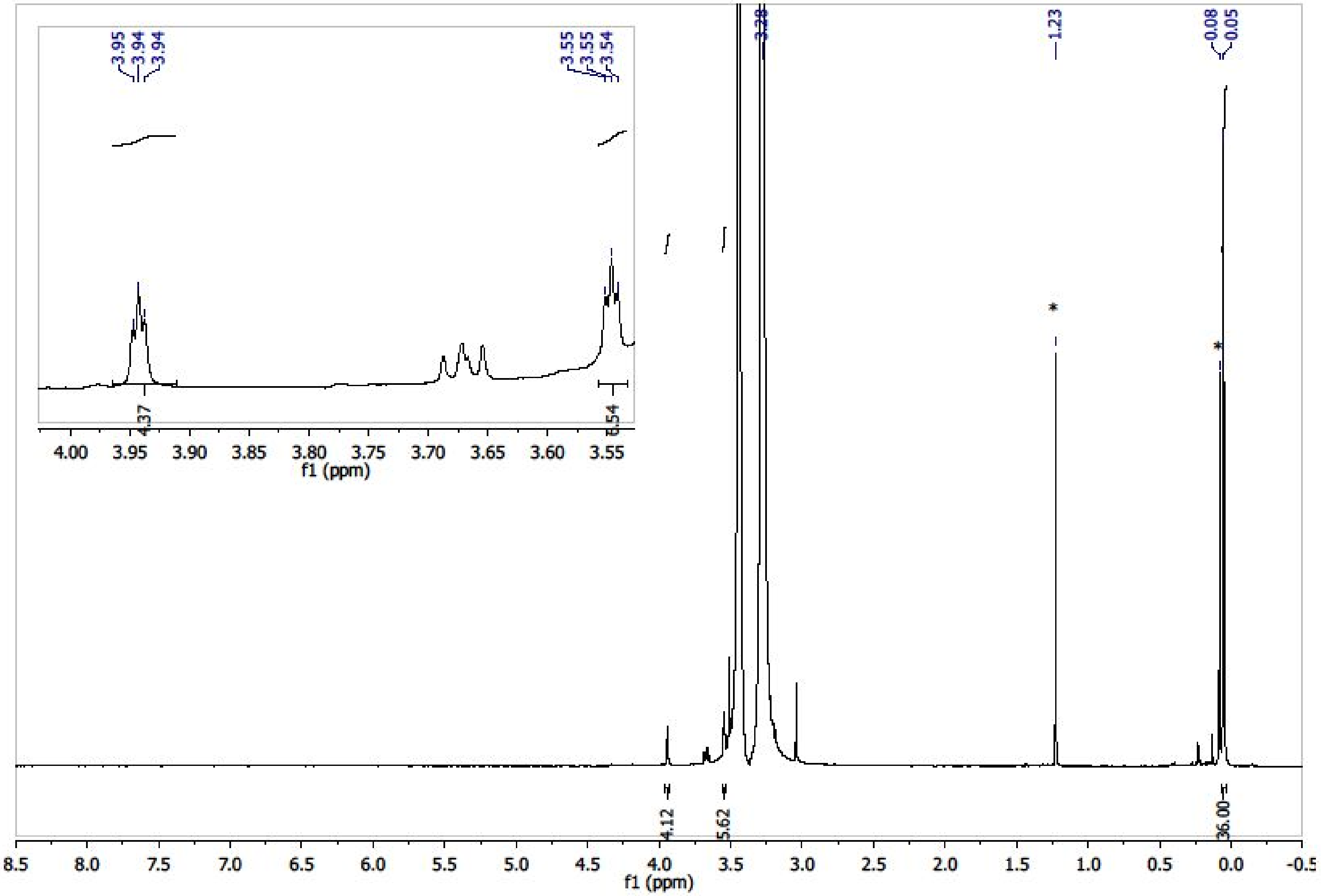

Figure S7. ^{1}H NMR spectrum of compound **6** in DME (* = ^{t}BuOSiMe$_3$)

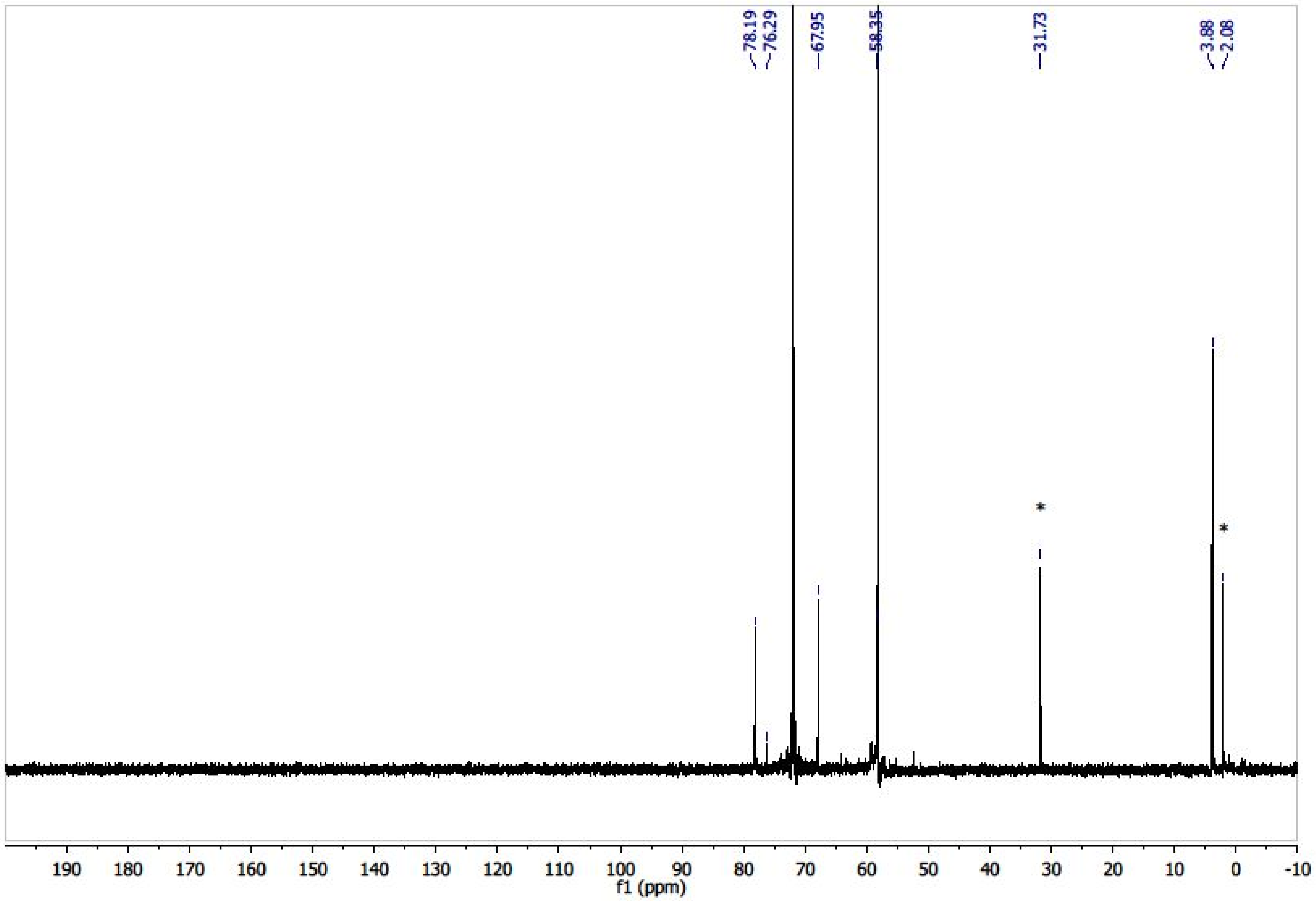

Figure S8. ^{13}C NMR spectrum of compound **6** in DME (* = ^{t}BuOSiMe$_3$)

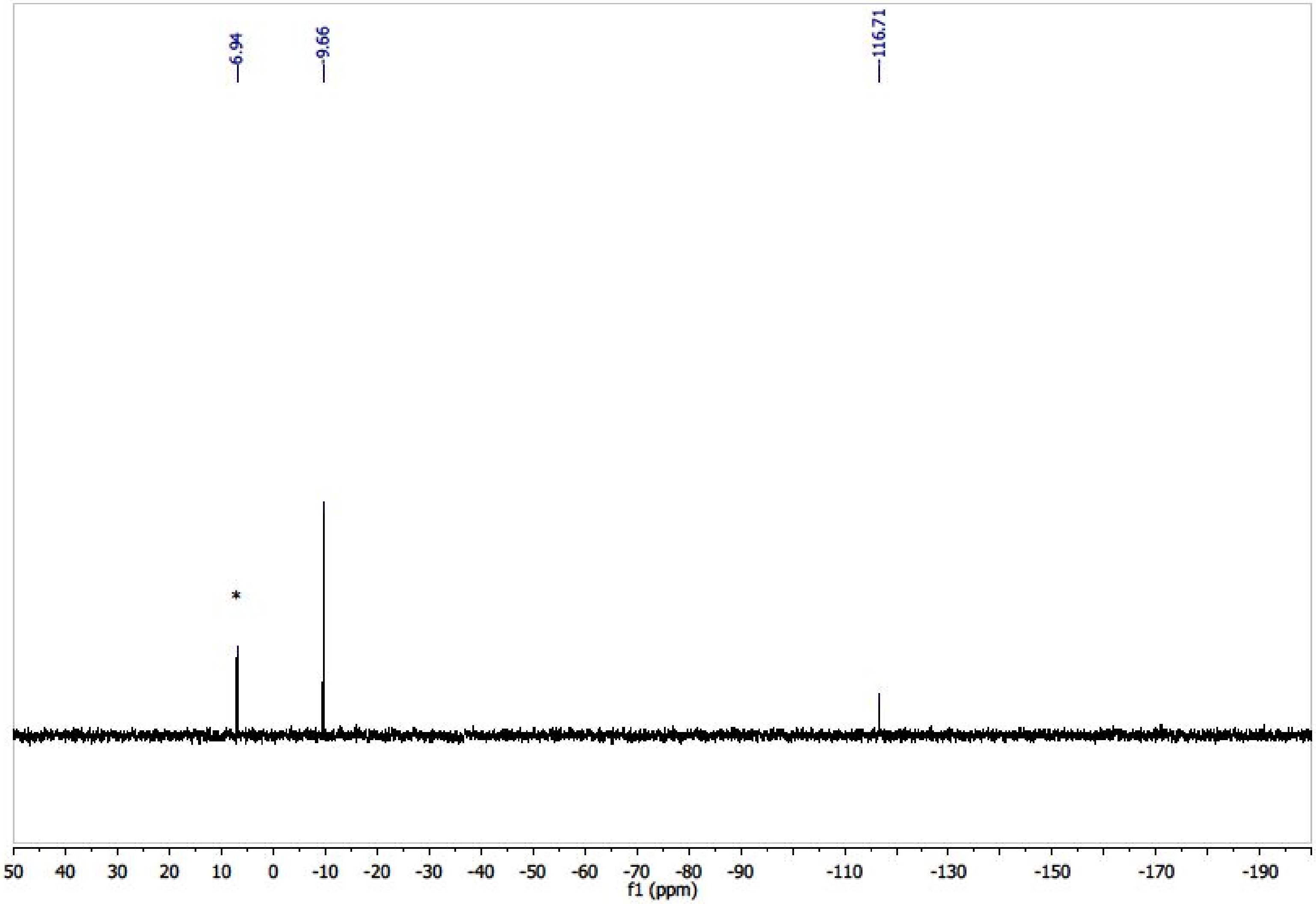

Figure S9. ^{29}Si INEPT NMR spectrum of compound **6** in DME (* = ^{t}BuOSiMe$_3$)

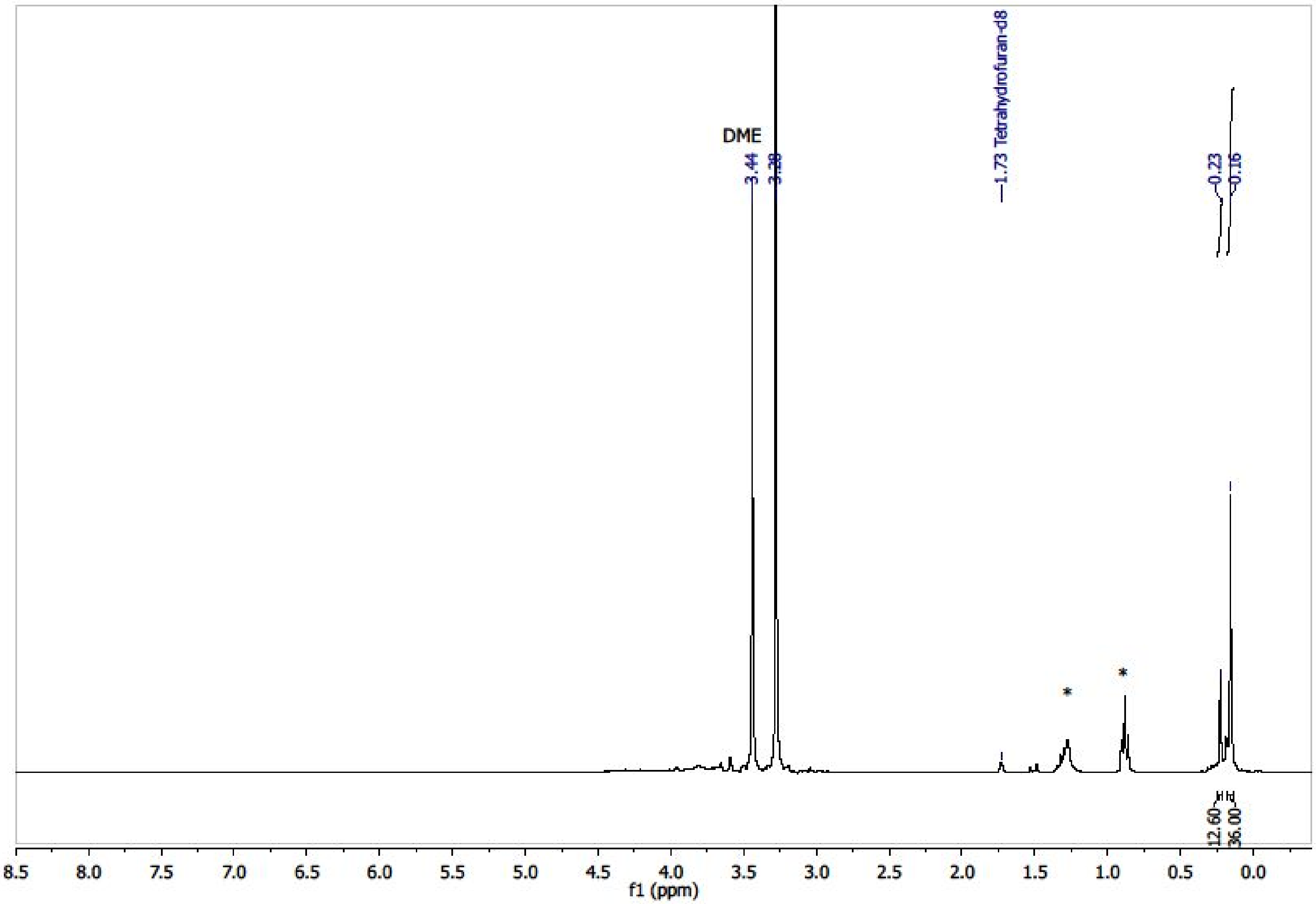

Figure S10. ^{1}H NMR spectrum of compound **7** in DME/THF-d_8 (* = residual pentane)

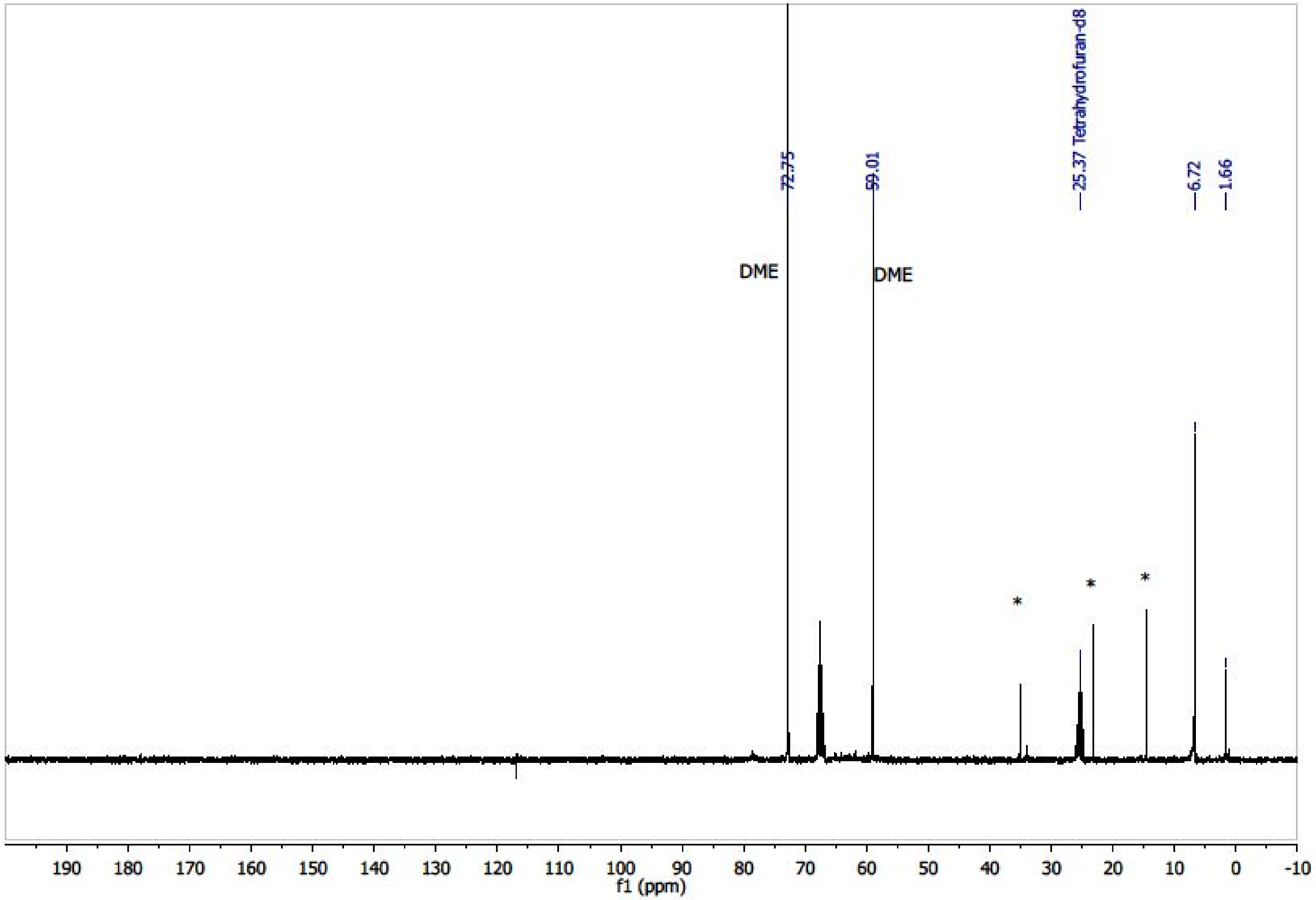

Figure S11. ^{13}C NMR spectrum of compound **7** in DME/THF-d_8 (* = residual pentane)

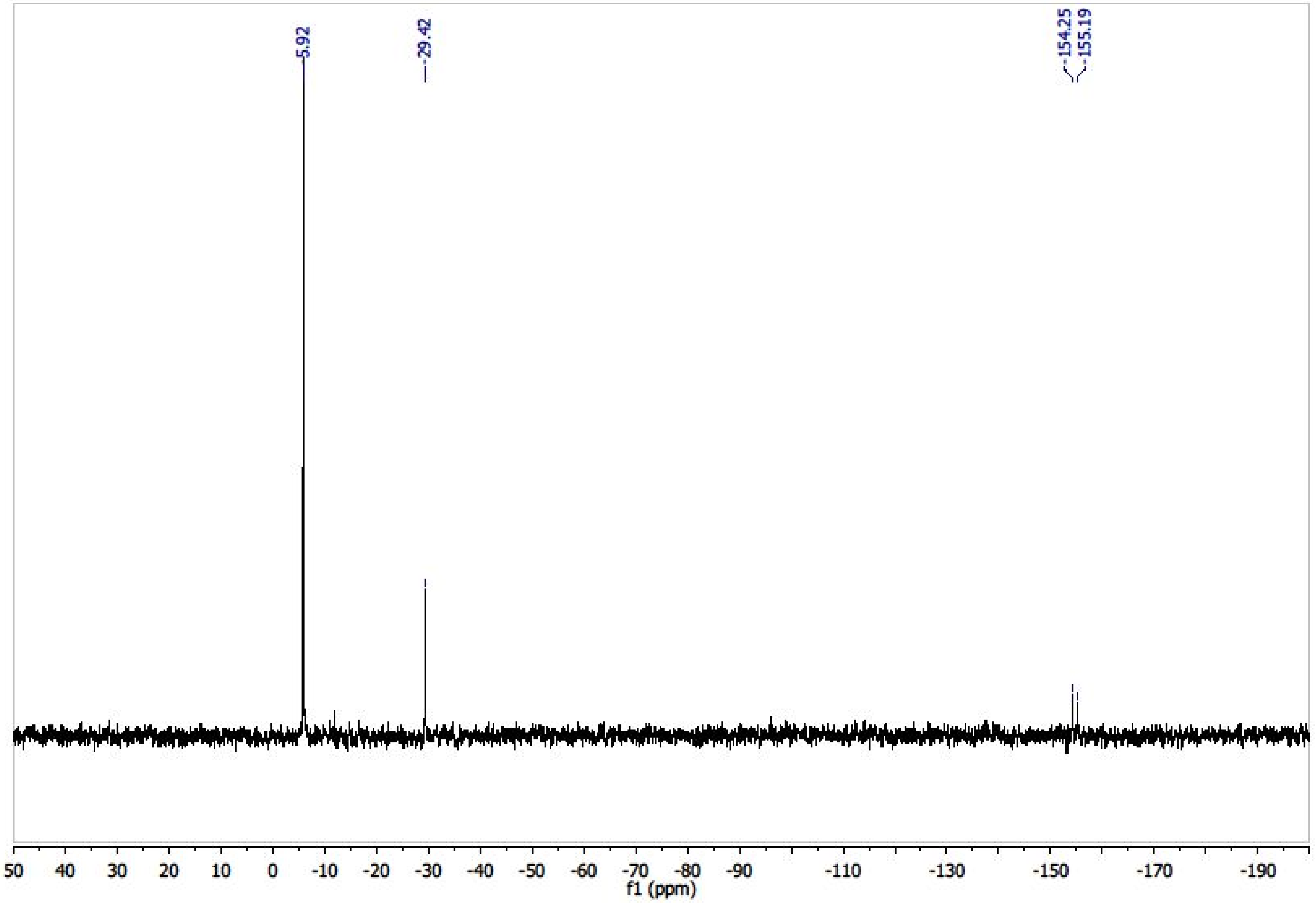

Figure S12. ^{29}Si INEPT NMR spectrum of compound **7** in DME/THF-d_8

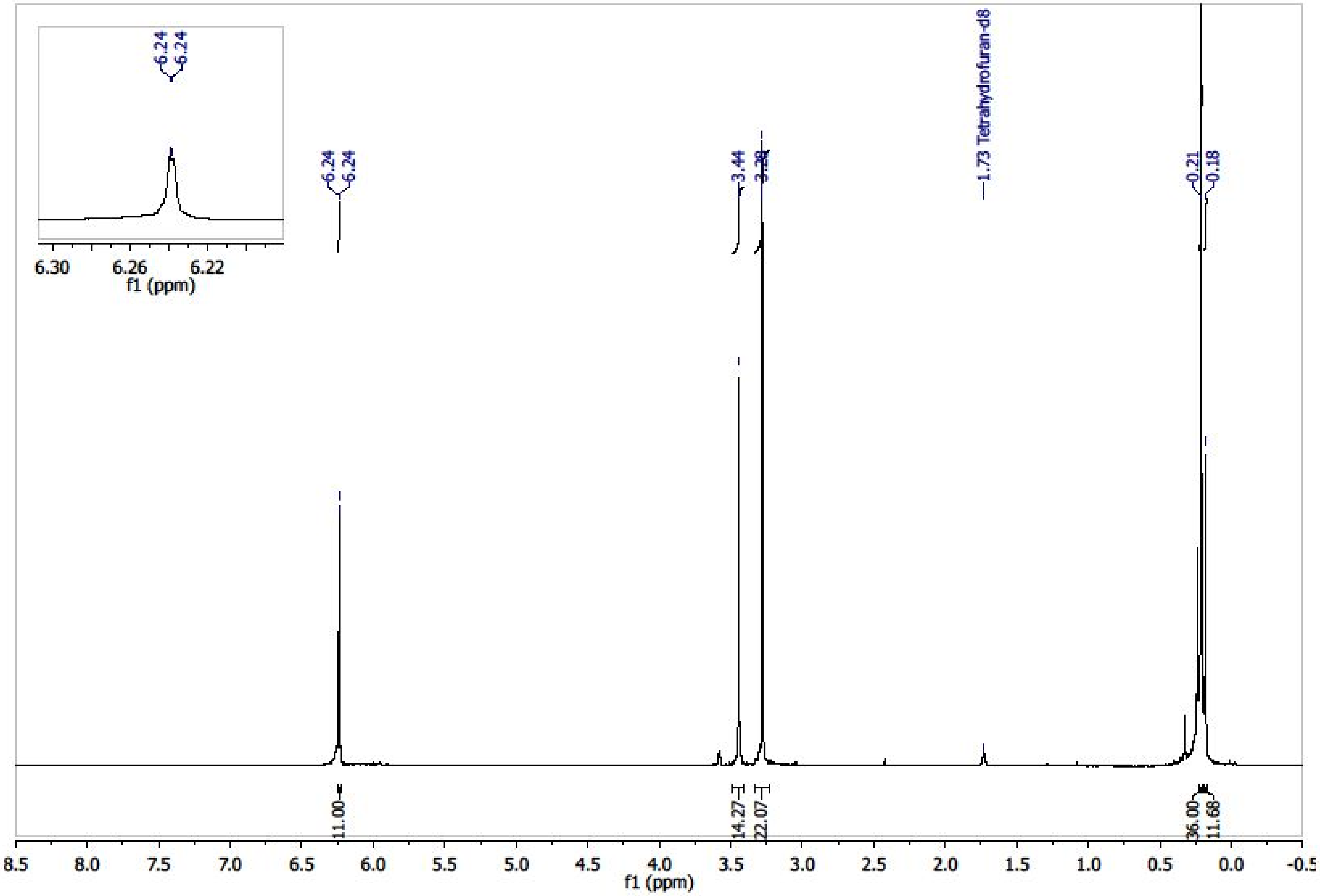

Figure S13. ^{1}H NMR spectrum of compound **8** in THF-d_8

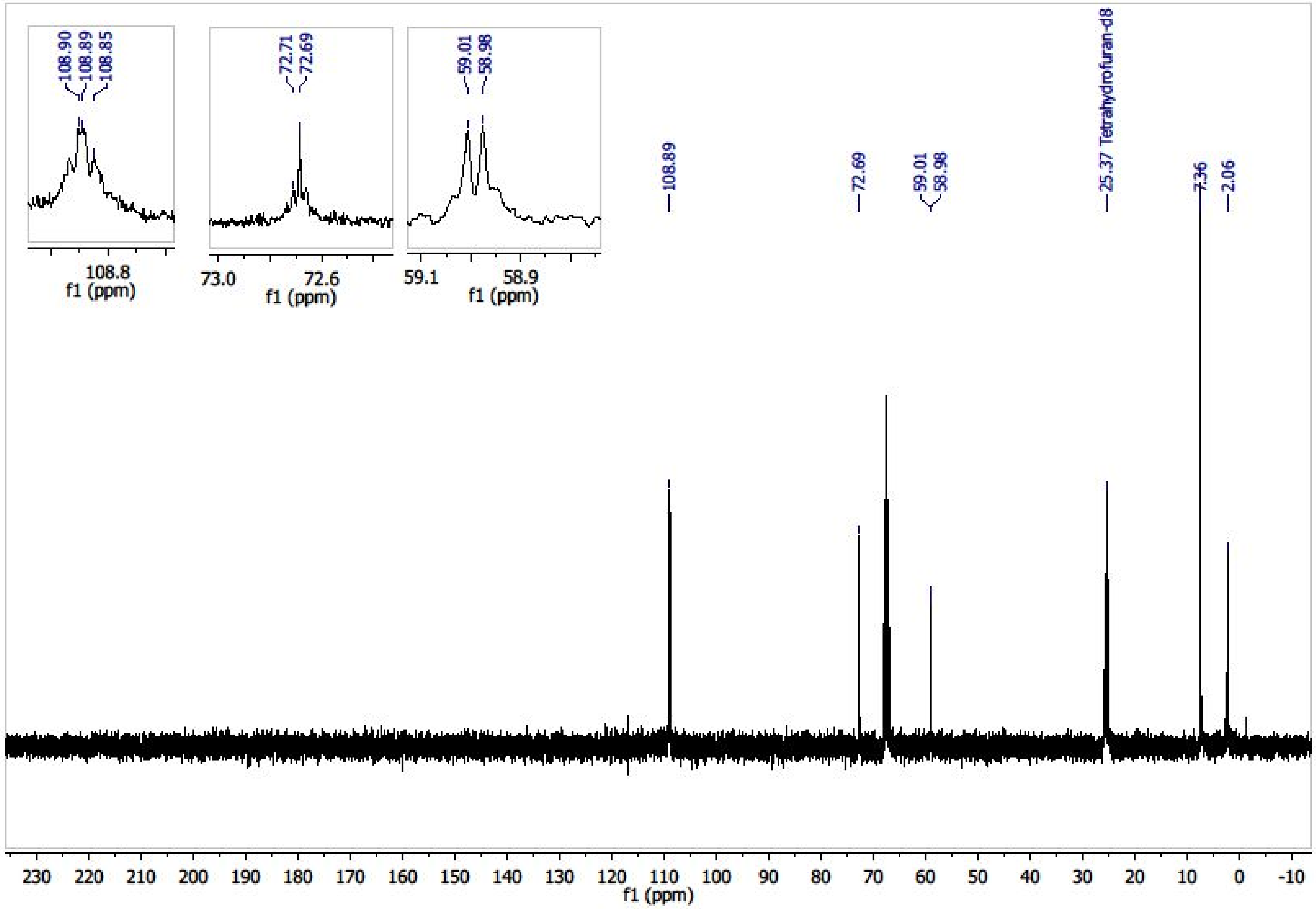

Figure S14. ^{13}C NMR spectrum of compound **8** in THF-d_8

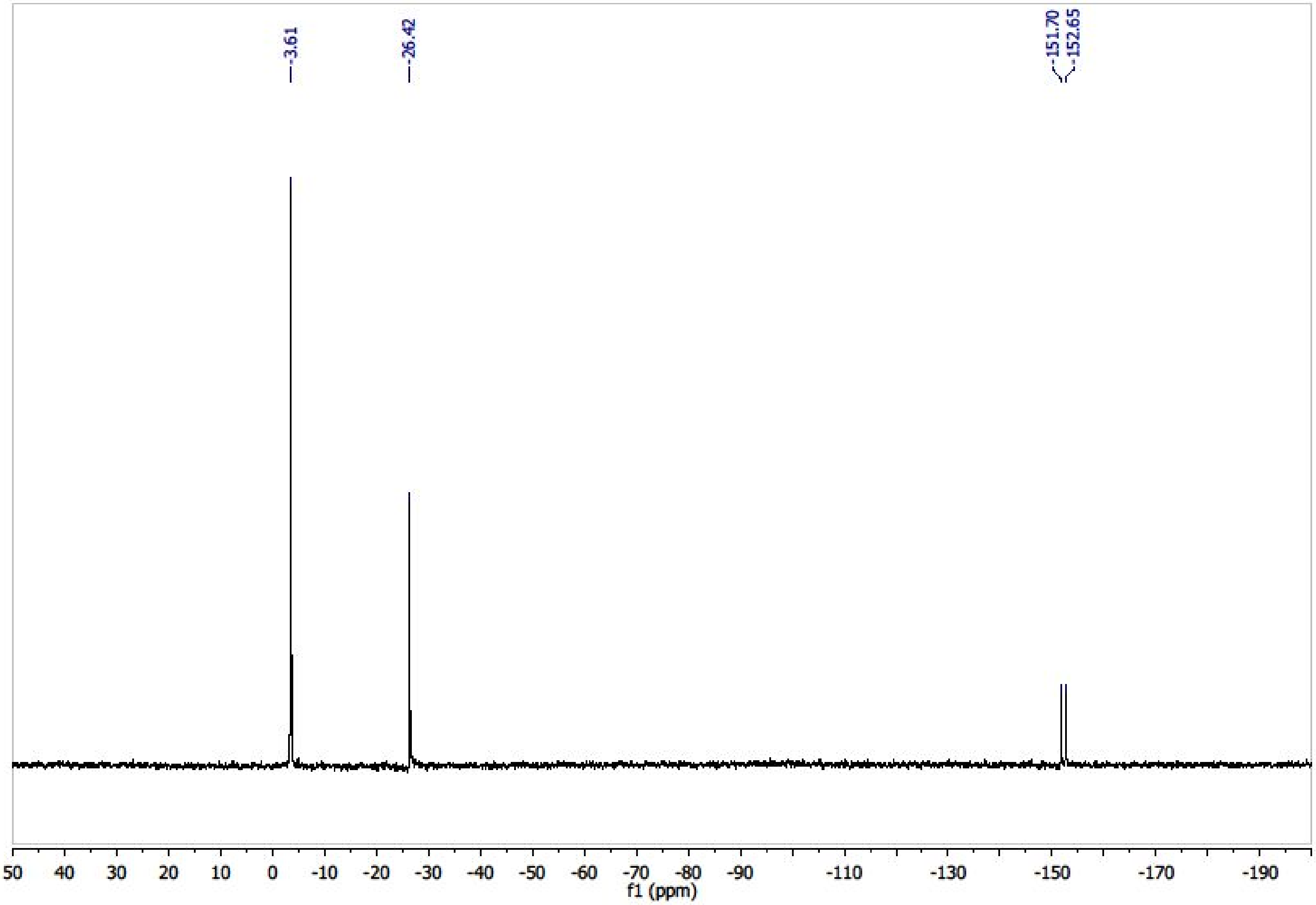

Figure S15. ^{29}Si INEPT NMR spectrum of compound **8** in THF-d_8

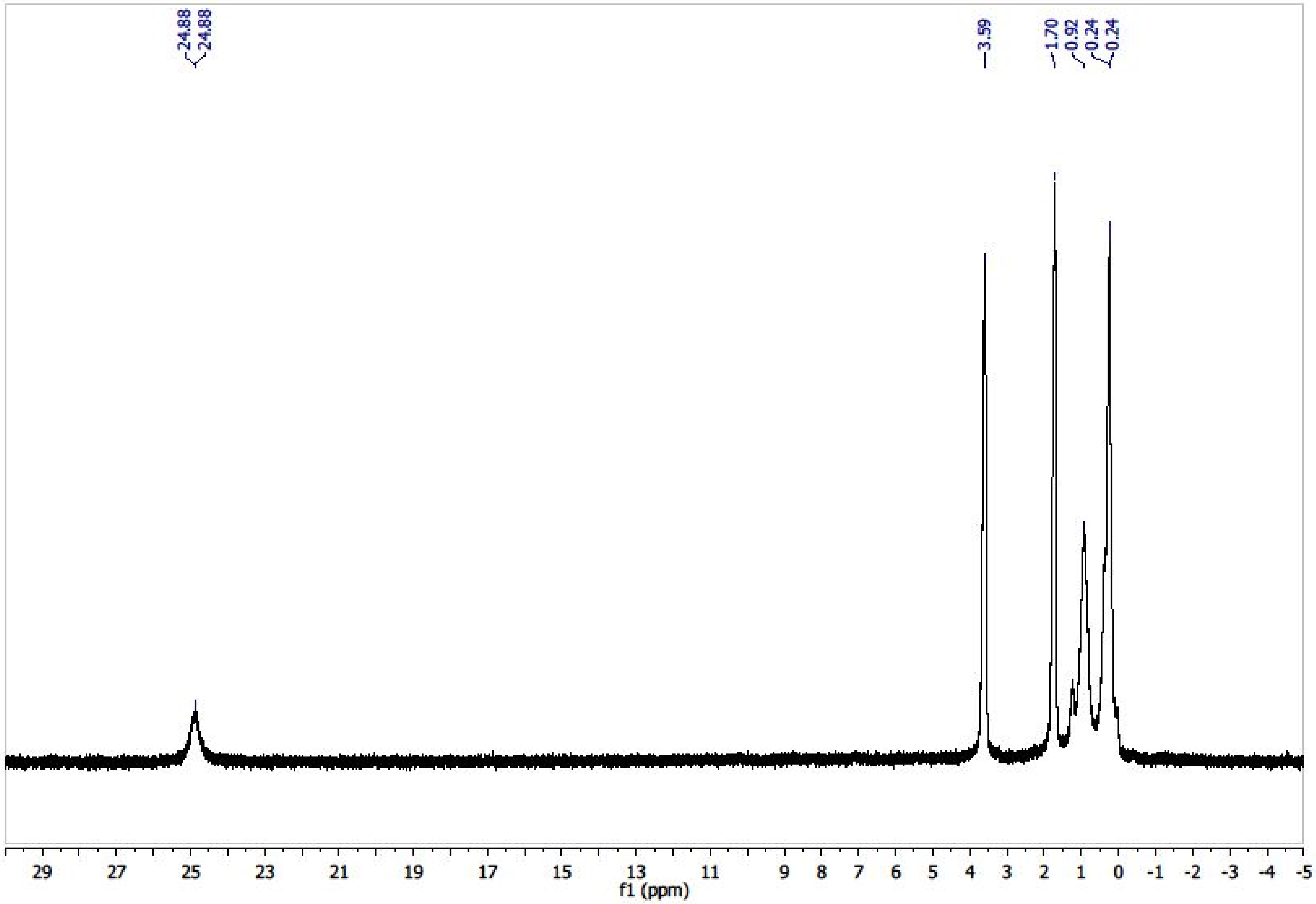

Figure S16. ^{1}H NMR spectrum of compound **9** in THF-d_8

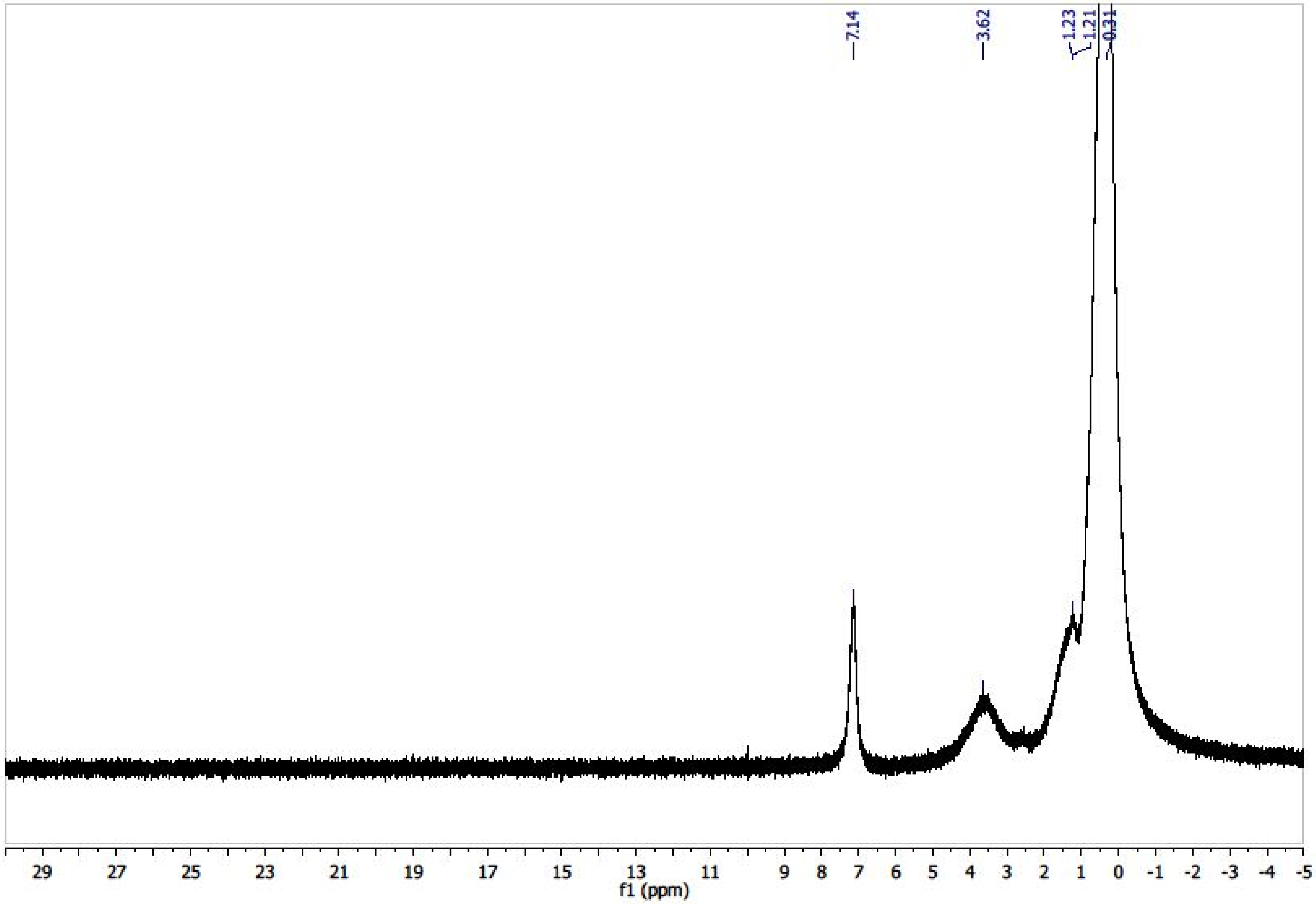

Figure S17. ^{1}H NMR spectrum of compound **10** in C_6D_6
